# Supplementary material for: Behavioural compatibility, not fear, best predicts the looking patterns of chacma baboons
Source: Commun Biol. 2024 Aug 12;7:980. doi: 10.1038/s42003-024-06657-w (PMC11319805; doi:10.1038/s42003-024-06657-w)
Supplement: Supplementary file 2 — Supplementary Information [file 42003_2024_6657_MOESM2_ESM.pdf]

Supplementary information

Table S1. Key drivers of risk in group living animals and the relevant type of vigilance that may be used to monitor or avoid threats.

Driver of vigilance	Risk theory	Type of vigilance	Example
**Presence of an extra-group threat**	Risk higher in presence of threat	Reactionary vigilance (antipredator/extragroup)	1
**Within-group conflict**	Risk higher during conflict	Reactionary vigilance (within-group)	2[ᵻ]
**Spatial cohesion (external threats)**	Risk increases as group cohesion decreases	Pre-emptive vigilance (antipredator/extragroup)	3
**Spatial cohesion (within-group threats)**	Risk increases as group cohesion increases	Within-group (social) vigilance	4
**Spatial position (external threats)**	Risk increases at the periphery of a group	Pre-emptive vigilance (antipredator/extragroup)	5
**Spatial position (conspecific monitoring hypothesis)**	Risk higher in the centre/ at the front of a group	Within-group (social) vigilance	6
**Visibility (visual obstruction hypothesis)**	Risk increases as visibility decreases	Pre-emptive vigilance (all threats)	7
**Visibility (protective cover hypothesis)**	Risk increases as visibility increases	Pre-emptive vigilance (all threats)	8
**Distance to cover (protective hypothesis)**	Risk increases with distance to cover	Pre-emptive vigilance (antipredator)	9
**Distance to cover (obstruction hypothesis)**	Risk decreases with distance to cover	Pre-emptive vigilance (antipredator)	10
**Landscape of fear (external threats)**	Risk increases in high-risk areas	Pre-emptive vigilance (all external threats)	11
**Range overlap (foreign conspecific groups)**	Risk increases in areas of high overlap	Pre-emptive vigilance (foreign conspecifics)	12
**Boundary areas (external threats)**	Risk increases in areas close to edge of home-range	Pre-emptive vigilance (all external threats)	3
**Dominance**	Risk higher for subordinates	Within-group (social) vigilance	13
**Identity of neighbours**	Risk increases with proximity of threatening neighbours	Within-group (social) vigilance	14
**Time of day (predation risk)**	Risk increases in lower light levels	Pre-emptive vigilance (antipredator)	15
**Time of day (energetic demands)**	Vigilance increases with increasing energy reserves	Pre-emptive vigilance (all threats)	16
**Body size (all threats)**	Risk higher for smaller individuals	Pre-emptive vigilance (all threats)	17
**Reproductive state (Gestation/lactation hinders escape ability)**	Risk increases with gestation/lactation period	Pre-emptive vigilance (all threats)	18
**Reproductive state (Gestation/lactation reduces energy levels)**	Vigilance decreases with increasing energetic demands	Pre-emptive vigilance (all threats)	19
**Maternal care (all threats)**	Risk higher for females with infants	Pre-emptive vigilance (all threats)	20
**Maternal care - infant age (all threats)**	Risk decreases as infant age increases	Pre-emptive vigilance (all threats)	21
**Maternal care - infant distance (all threats)**	Risk increases with distance to infant	Pre-emptive vigilance (all threats)	22
**Male consortship status (intra-sexual competition hypothesis)**	Risk higher when mate guarding	Pre-emptive and reactionary vigilance (conspecific)	23
**Observer distance (threat hypothesis)**	Risk increases with observer proximity	Pre-emptive vigilance (all threats*)	24
**Observer distance (human-shield hypothesis)**	Risk decreases with observer proximity	Pre-emptive vigilance (all threats*)	25
**Observer movement**	Risk higher when movement occurs	Reactionary vigilance (observer)	24

[ᵻ]Conflict between conspecifics draws visual attention for individuals not engaged in conflict, which can compete with antipredator vigilance. *Potential for habituated animals to exploit within-group tolerance differences, therefore, tolerant phenotypes could perceive less risk when in proximity to observers.

Text S1. Detailed model justifications

We fitted an intercept only model (see Main Text Table 1: *Model 1*) as this offered an important candidate model for comparison within each stack, if it yielded greater prediction accuracy than other models it would suggest the independent variables were poor predictors of looking.

Some of the most common themes explored in primate vigilance research are differences in vigilance use across sexes[3,23], ages[26–28], female reproductive states[21,26,29], and infant conspicuousness/proximity to mothers [21,22,30], and differences in vigilance use between different behaviours or activities[9,31–33]. Thus, our second model explored these hypotheses independent to other potential factors by including a variable for age-sex class (including distinctions between adult females without dependent infants, with their dependent infant in proximity, and with their dependent infant out of sight), and a variable for time spent engaged/not engaged. These models are described as our minimal models as both variables were also included in all other candidate models to account for their role in governing looking patterns. Time spent engaged (i.e., grooming, digging, picking fruit) was used as a predictor for all frequency of looking models, and time spent not engaged (i.e., resting, receiving grooming, handling food items) was used for all models using the duration response variable. For most hypotheses if time spent 'engaged' is high then the expectation is that frequency of looking bouts become the more risk-sensitive response variable. Whereas duration of looking bouts is a more risk-sensitive response variable when time spent 'not engaged' is high.

Spatial position and cohesion hypotheses can make competing predictions depending on whether threats are from internal or external group threats. To explore these hypotheses, we included candidate models exploring the interactive effect of number of neighbours within five meters and spatial position (i.e., central or peripheral) of the focal animal on both the frequency and duration of looking bouts (see Table 1: *Model 3*). Thus, both models (one for each response variable) explore the external threat hypothesis, i.e., risk is highest when an animal is peripheral and the group is sparse, and the conspecific risk hypothesis, i.e., risk is highest when central and the group is cohesive (see Table S1).

Primate vigilance research is made challenging by differing postural tendencies and handling abilities of each species, with evidence suggesting time spent using vigilance is tied to the ratio of search time and compatible handling time[34]. Some food items such as roots can require handling to clean dirt away before consumption, whilst certain fruits and seeds require a different form of manual processing where the teeth are used to crack open a hard casing or to peel off the skin of a fruit; as the eyes are not necessarily needed for the processing task, the animal may be able to briefly scan their surroundings whilst biting. These tasks may offer compatible or cost-free looking time[35], and looking behaviours may not need to be risk sensitive in these scenarios and could potentially also hold a positive correlation with looking bouts, i.e., foraging success could 'promote' the frequency of looking bouts for certain food items. Each response variable therefore included a model (see Table 1: *Model 4*) to specifically explore the association between looking patterns and the predominant food item consumed during the focal observation, and additionally, the relationship between feeding rate (i.e., number of bites or items placed in the focal animal's mouth) and both looking variables.

Each response variable also included a model (*Model 5*) designed to explore whether specific behaviours are associated with consistent patterns of looking behaviours, e.g., as handling time increases (regardless of the specific item) the duration or frequency of looking may increase. Primates can also be engaged in tasks such as auto-grooming and allogrooming which may lead to

decreased investment in looking or vigilance as monitoring could be shared between partners or could have a calming effect on both individuals[33]. As baboons utilise a range of postures[36], we also included the focal animal's predominant posture during the focal observation within the specific behaviours models, i.e., sitting, laying, or standing (quadrupedal or bipedal). Although models 4 and 5 do not explore specific risk drivers of looking, both are important alternative hypotheses for understanding the scenarios that promote or constrain looking generally. For example, animals may not need to be pre-emptively vigilant if they readily utilise cost-free or compatible moments of looking time during certain behaviours[37].

Despite several studies noting the important differences between induced and routine vigilance [35,38] or pre-emptive and reactionary vigilance[26], many primate research studies overlook this key distinction[37]. We included a set of candidate models (Table 1: *Models 6 – 14*) that describe whether certain events are ongoing and the time since these events ended. These events included non-threatening within-group stimuli (e.g., copulations, female 'lost' calls), potentially threatening within-group stimuli (e.g., male wahoos and within-group aggressions), passive and active heterospecific encounters (e.g., an animal walking past versus charging group members), group-wide alarm episodes (e.g., during predator encounters), encounters with domestic dogs, and encounters with other groups of baboons. Non-threatening events can still draw visual attention and therefore potentially lead to increased looking, as such they are important to explore within a looking framework as overlooking them could lead to important information being missed. For example, copulations may draw the visual attention of male competitors.

The counterpart to reactionary vigilance, pre-emptive vigilance, has received more attention in primate research[37]. Although risk is typically perceived to be from predators, for group-living species experiencing contest competition for resources, within-group threats may pose a greater risk to fitness[39]. Although social vigilance has received a lot of attention in primate vigilance research, numerous variables have been used as proxies for threats, including distance to nearest neighbours[40], number of neighbours[4,32], rank of neighbours[13,31], and relationship to neighbours[41,42]. Model 15 (see Table 1) explored the patterns of looking behaviours in response to changes in the number of social threats within five meters of the focal animal. We considered social threats to be any individual ranked higher than the focal animal who was not part of their social 'clique' (as identified from a grooming data).

Our next set of models (*Models 16 – 20*) explored the remaining pre-emptive vigilance scenarios. This included the spatial likelihood of encountering leopards (*Panthera pardus*) (see [43,44]), the likelihood of encountering other groups of baboons[43,45], home-range familiarity[3], and habitat type. We explored the effect of home range familiarity on looking using a continuous and a categorical variable. Habitat type allowed exploration into how the baboons may perceive fear differently according to specific human infrastructures such as farms and researcher camps. For example, camp settings may offer baboons safety from leopards, whilst farms may reduce leopard risk at the cost of proximity with unfamiliar and potentially threatening humans. This additionally allowed exploration into whether they perceive risk differently across broadly classified vegetation types including grassland, bushland, woodland, and forests, as leopards are known to preferentially utilise densely vegetated areas at Lajuma[44].

Primate vigilance literature has typically focused on predators and conspecifics as the major drivers of vigilance in primate groups, with little work exploring the role of humans (although see [46]). An implicit assumption in research using direct observations of habituated animals is that the study subjects no longer fear observers and view them as neutral stimuli; however, our previous results challenged that these assumptions applied to our study group[47]. We also found an individual-level

tolerance trait had emerged, whereby the study animal's did not appear equally tolerant to observer approaches[47]. These individual tolerance levels were consistent across two observers differing in familiarity and across a range of scenarios, including after a predation event from a leopard[48]. When researchers were within 4.5 meters of focal subjects, we also found a positive association between tolerance and how often an individual occurred as a neighbour to focal animals, i.e., very intolerant animals avoided proximity to researchers[49]. In our final model (Table 1: *Model 21*), we explore whether interactions between tolerance level and observer distance, and tolerance and observer movement, during observations influenced looking patterns.

Visibility was included as a predictor in several models (see Table 1: *Models 6, 9, 10, and 12 – 20*) to account for the competing predictions of the protective cover hypothesis and the visual obstruction hypotheses (see Table 1), and therefore was not used in minimal models, or models exploring non-threatening stimuli. As with visibility, the dominance rank of the focal individual was also included as a predictor in all models exploring risk hypotheses to account for possible rank effects on looking and vigilance behaviours[50–52].

Model summary results and figures

Table S2. Intercept summary results for frequency response variable. Date and individual identity fitted as crossed group-level (i.e., random) effects.

	Estimate	Est.Error	l-95% CI	u-95% CI	Rhat	Bulk_ESS	Tail_ESS
Intercept	-2.36	0.03	-2.42	-2.3	1	1100	1740
Date							
sd(Intercept)	0.16	0.02	0.13	0.2	1	1626	2596
ID							
sd(Intercept)	0.18	0.02	0.15	0.23	1	1212	1913

Table S3. Intercept summary results for duration response variable. Date and individual identity fitted as crossed group-level (i.e., random) effects.

	Estimate	Est.Error	l-95% CI	u-95% CI	Rhat	Bulk_ESS	Tail_ESS
Intercept	-95.69	17.69	-137.4	-68.99	1	2135	2031
Date							
sd(Intercept)	16.09	3.43	10.42	23.7	1	1985	2353
ID							
sd(Intercept)	24.21	4.76	16.71	35.16	1	2201	2443
Family							
sigma	34.58	3.34	29.25	42.02	1	2219	1836

Minimal models

Table S4. Frequency model summary with age-sex class and time spent engaged as population-level effects and date and individual identity fitted as crossed group-level (i.e., random) effects.

	Estimate	Est.Error	l-95% CI	u-95% CI	Rhat	Bulk_ESS	Tail_ESS
Intercept	-2.09	0.06	-2.2	-1.98	1.01	637	1166
Time spent engaged	-0.01	0	-0.01	-0.01	1.00	4051	2939
Adolescent males	-0.46	0.1	-0.66	-0.26	1.00	1037	1996
Adult females	-0.19	0.06	-0.31	-0.07	1.00	876	1769
Adult females (Infant contact)	-0.22	0.07	-0.35	-0.09	1.00	1151	1806
Adult females (Infant OS)	-0.08	0.12	-0.31	0.15	1.00	2520	2779
Adult males	-0.42	0.08	-0.58	-0.27	1.00	943	1544
Juvenile-1 females	0.07	0.08	-0.08	0.22	1.00	1192	1822
Juvenile-1 males	-0.08	0.08	-0.24	0.07	1.00	986	1510
Juvenile-2 females	-0.06	0.06	-0.18	0.06	1.00	1505	2440
Juvenile-2 males	-0.13	0.07	-0.26	0.01	1.00	831	1726
Juvenile-3 males	-0.24	0.08	-0.41	-0.08	1.00	859	1454
Date							
sd(Intercept)	0.17	0.02	0.13	0.21	1.00	1306	2427
ID							
sd(Intercept)	0.13	0.02	0.1	0.17	1.01	1033	2280

Table S5. Duration model summary with age-sex class and time spent not engaged as population-level effects and date and individual identity fitted as crossed group-level (i.e., random) effects.

	Estimate	Est.Error	l-95% CI	u-95% CI	Rhat	Bulk_ESS	Tail_ESS
Intercept	-61.28	2.08	-65.35	-57.41	1.00	1405	2274
Adolescent males	3.05	1.88	-0.59	6.74	1.00	1804	2718
Adult females	0.69	1.22	-1.7	3.15	1.00	1118	1980
Adult females (Infant contact)	1.71	1.43	-1.12	4.49	1.00	1341	2321
Adult females (Infant OS)	2.41	3.34	-4.27	8.9	1.00	3890	2896
Adult males	3.52	1.39	0.81	6.24	1.00	1247	1915
Juvenile-1 females	-2.18	1.63	-5.36	0.94	1.00	1663	2515
Juvenile-1 males	-1.87	1.54	-4.75	1.29	1.00	1604	2443
Juvenile-2 females	-0.13	1.48	-3.06	2.77	1.00	1593	2633
Juvenile-2 males	3.07	1.35	0.48	5.71	1.00	1325	2279
Juvenile-3 males	2.3	1.56	-0.78	5.37	1.00	1388	1908
Time spent not engaged	1.78	0.06	1.66	1.9	1.00	2187	2852
Date							
sd(Intercept)	2.15	0.38	1.44	2.92	1.00	1786	2250
ID							
sd(Intercept)	1.27	0.47	0.25	2.13	1.00	745	465
Family							
sigma	10.56	0.25	10.08	11.08	1.00	2132	2793

Interaction between spatial position and cohesion

Table S6. Summary results for frequency model exploring the effect of the interaction between spatial position (central/peripheral) and spatial cohesion (number of neighbours within 5 meters). The remainder of the population-level and group-level factors are the same as the minimal model.

	Estimate	Est.Error	l-95% CI	u-95% CI	Rhat	Bulk_ESS	Tail_ESS
Intercept	-2.05	0.06	-2.17	-1.93	1.00	574	1179
Adolescent males	-0.44	0.1	-0.64	-0.25	1.00	1336	2508
Adult females	-0.19	0.06	-0.31	-0.08	1.00	583	1201
Adult females (Infant contact)	-0.19	0.07	-0.32	-0.05	1.00	818	1201
Adult females (Infant OS)	-0.08	0.12	-0.3	0.14	1.00	1979	2497
Adult males	-0.41	0.08	-0.56	-0.27	1.00	1033	2053
Juvenile-1 females	0.08	0.07	-0.07	0.23	1.00	889	1945
Juvenile-1 males	-0.04	0.08	-0.2	0.12	1.00	911	1703
Juvenile-2 females	-0.05	0.06	-0.18	0.07	1.00	1151	2068
Juvenile-2 males	-0.11	0.07	-0.25	0.02	1.00	831	1489
Juvenile-3 males	-0.22	0.08	-0.38	-0.05	1.00	880	1408
Spatial position (Peripheral)	0.08	0.03	0.02	0.14	1.00	2724	2755
Number of neighbours (5m)	-0.04	0.01	-0.05	-0.02	1.00	3344	3139
Time spent engaged	-0.01	0	-0.01	-0.01	1.00	4248	3574
Peripheral : Number of neighbours	-0.02	0.01	-0.04	0.01	1.00	2650	2739
Date							
sd(Intercept)	0.16	0.02	0.13	0.2	1.00	1410	2233
ID							
sd(Intercept)	0.13	0.02	0.1	0.16	1.00	1661	2309

Table S7. Summary results for duration model exploring the effect of the interaction between spatial position (central/peripheral) and spatial cohesion (number of neighbours within 5 meters). The remainder of the population-level and group-level factors are the same as the minimal model.

	Estimate	Est.Error	l-95% CI	u-95% CI	Rhat	Bulk_ESS	Tail_ESS
Intercept	-61.22	2.16	-65.57	-57.21	1.00	1408	1396
Adolescent males	3.12	1.83	-0.37	6.67	1.00	1964	2507
Adult females	0.68	1.19	-1.71	2.99	1.01	1038	1648
Adult females (Infant contact)	1.81	1.45	-1.11	4.68	1.00	1286	2016
Adult females (Infant OS)	2.41	3.38	-4.54	8.77	1.00	3621	2719
Adult males	3.6	1.35	0.85	6.28	1.00	1162	1812
Juvenile-1 females	-2.1	1.6	-5.33	1.17	1.00	1575	1992
Juvenile-1 males	-1.79	1.52	-4.67	1.19	1.00	1501	2109
Juvenile-2 females	-0.09	1.47	-3.04	2.78	1.00	1574	2333
Juvenile-2 males	3.1	1.35	0.33	5.67	1.00	1362	1943
Juvenile-3 males	2.35	1.57	-0.72	5.42	1.00	1449	2142
Spatial position (Peripheral)	0.14	0.76	-1.34	1.61	1.00	3634	3579
Number of neighbours (5m)	-0.1	0.16	-0.42	0.23	1.00	3926	3062
Time spent engaged	1.78	0.06	1.67	1.91	1.00	1949	2220
Peripheral : Number of neighbours	-0.15	0.31	-0.76	0.43	1.00	3618	3007
Date							
sd(Intercept)	2.11	0.38	1.4	2.89	1.00	1318	2281
ID							
sd(Intercept)	1.25	0.5	0.14	2.16	1.00	735	490
Family							
sigma	10.58	0.26	10.09	11.09	1.00	1905	2563

Table S8. Summary results for frequency model exploring the effect of feeding rate and predominant food item on looking behaviours. The remainder of the population-level and group-level factors are the same as the minimal model. *Acacia/Senegalia ataxacantha* seeds pods and adolescent females embedded within the intercept.

	Estimate	Est.Error	l-95% CI	u-95% CI	Rhat	Bulk_ESS	Tail_ESS
Intercept	-2.01	0.07	-2.15	-1.86	1.00	1189	2005
Amount Eaten	0.02	0	0.01	0.02	1.00	6562	3501
Acacia/Vachellia karoo seed pods	-0.05	0.06	-0.18	0.07	1.00	1312	2047
Acacia/Vachellia sieberiana seed pods	0.73	0.08	0.57	0.89	1.00	1585	2588
Grass corm	0.23	0.11	0.02	0.45	1.00	2222	2840
Dichrostachys cinerea seed pods	0.35	0.07	0.22	0.48	1.00	1334	2506
Fruit (Small)	0.01	0.07	-0.14	0.14	1.00	1335	2268
Fruit (Large)	0.3	0.08	0.14	0.47	1.00	1708	2959
Grass blades	0.2	0.06	0.09	0.32	1.00	1107	2465
Grass seeds	0.15	0.08	0	0.3	1.00	1722	2455
Inverterbates	0.03	0.09	-0.15	0.2	1.00	1904	2771
Leaves	0.13	0.1	-0.06	0.31	1.00	2521	2581
No food eaten	-0.57	0.06	-0.68	-0.46	1.00	955	1894
Other	0.14	0.11	-0.07	0.35	1.00	3160	2976
Roots	0.18	0.06	0.07	0.29	1.00	936	1804
Succulent leaves	0.41	0.11	0.2	0.62	1.00	2293	2894
Unknown	0.07	0.07	-0.07	0.21	1.00	1358	1898
Seeds in leaf litter	0.18	0.07	0.04	0.32	1.00	1544	2167
Ziziphus mucronata fruit	0.24	0.1	0.04	0.43	1.00	2413	2719
Ziziphus mucronata seed	0.26	0.09	0.09	0.44	1.00	2198	2599
Adolescent males	-0.29	0.1	-0.47	-0.1	1.00	2322	2915
Adult females	-0.12	0.05	-0.22	-0.01	1.00	1479	2672
Adult females (Infant contact)	-0.1	0.06	-0.22	0.02	1.00	1985	2876
Adult females (Infant OS)	-0.12	0.12	-0.36	0.1	1.00	4130	3059
Adult males	-0.26	0.07	-0.4	-0.12	1.00	1562	2414
Juvenile-1 females	0.06	0.07	-0.07	0.2	1.00	1899	2346
Juvenile-1 males	-0.08	0.07	-0.22	0.06	1.00	1862	2546
Juvenile-2 females	-0.05	0.06	-0.17	0.06	1.00	2148	2553
Juvenile-2 males	-0.09	0.06	-0.21	0.03	1.00	1779	2403
Juvenile-3 males	-0.21	0.07	-0.35	-0.06	1.00	1950	2557
Time spent engaged	-0.02	0	-0.02	-0.02	1.00	5026	3978

Table S9. Summary results for duration model exploring the effect of feeding rate and predominant food item on looking behaviours. The remainder of the population-level and group-level factors are the same as the minimal model. *Acacia/Senegalia ataxacantha* seeds pods and adolescent females embedded within the intercept.

	Estimate	Est.Error	l-95% CI	u-95% CI	Rhat	Bulk_ESS	Tail_ESS
Intercept	-49.79	2.17	-54.05	-45.58	1.01	1207	1906
Amount Eaten	-0.45	0.07	-0.59	-0.32	1.00	4402	3015
Acacia/Vachellia karoo seed pods	-1.84	2.11	-5.93	2.48	1.00	1456	2203
Acacia/Vachellia sieberiana seed pods	-4.87	2.75	-10.37	0.33	1.00	1653	2284
Grass corm	-8.32	3.41	-14.92	-1.77	1.00	2825	2761
Dichrostachys cinerea seed pods	-10.03	2.02	-14.06	-6.12	1.00	1222	2386
Fruit (Small)	1.16	2.03	-2.88	5.09	1.00	1117	2081
Fruit (Large)	-0.22	2.18	-4.43	4.08	1.00	1524	2452
Grass blades	8.31	1.71	4.99	11.75	1.00	875	1767
Grass seeds	4.67	2.13	0.44	8.97	1.00	1253	1945
Inverterbates	0.34	2.34	-4.23	4.87	1.00	1518	2399
Leaves	7.99	2.74	2.61	13.29	1.00	1819	2167
No food eaten	0.75	1.51	-2.15	3.7	1.00	809	1486
Other	-2.51	2.8	-8.26	2.81	1.00	1819	2381
Roots	-8.66	1.63	-11.87	-5.47	1.00	883	1544
Succulent leaves	-4.7	3.21	-11.06	1.53	1.00	2028	2698
Unknown	-1.55	1.8	-5.05	2	1.00	1043	1977
Seeds in leaf litter	-1.13	2.04	-5.25	2.86	1.00	1125	1890
Ziziphus mucronata fruit	-4.29	2.56	-9.31	0.69	1.00	1722	2386
Ziziphus mucronata seed	-4.26	2.32	-8.93	0.15	1.00	1493	2624
Adolescent males	1.3	1.6	-1.89	4.42	1.00	1940	2494
Adult females	-0.12	1.07	-2.27	1.95	1.00	1238	1670
Adult females (Infant contact)	0.15	1.26	-2.33	2.65	1.00	1553	2343
Adult females (Infant OS)	2.37	2.86	-3.36	7.86	1.00	3987	3215
Adult males	1.61	1.2	-0.74	3.95	1.00	1212	2172
Juvenile-1 females	-1.88	1.4	-4.59	0.87	1.00	1602	2518
Juvenile-1 males	-1.93	1.36	-4.67	0.71	1.00	1456	2232
Juvenile-2 females	0.02	1.3	-2.58	2.55	1.00	1851	2452
Juvenile-2 males	2.12	1.18	-0.24	4.39	1.00	1228	1959
Juvenile-3 males	1.77	1.39	-1.02	4.36	1.00	1510	2480
Time spent engaged	1.47	0.05	1.38	1.57	1.00	2486	2737
Date							
sd(Intercept)	1.37	0.35	0.61	2.02	1.00	1157	1250
ID							
sd(Intercept)	1.19	0.42	0.23	1.95	1.00	747	527
Family							
sigma	9.29	0.21	8.91	9.73	1.00	2370	2788

Table S10. Model summary results for specific behaviours predicting the frequency and total duration of looking behaviours. Upper and lower 95% credible intervals are shown within parentheses. Bold text highlight parameter estimates where the Cis did not overlap or include zero. All R-hat (Gelman-Rubin convergence diagnostic) were less than 1.01 suggesting accurate estimates of the posterior distribution. In all cases the bulk estimated sample size (bulk_ESS) was greater than 100 times the number of chains (i.e., bulk_ESS > 400) indicating the mean was efficiently sampled in all cases.

	Frequency					Duration				
	Estimate and CIs	Est.Error	Rhat	Bulk_ESS	Tail_ESS	Estimate and CIs	Est.Error	Rhat	Bulk_ESS	Tail_ESS
Intercept	**-2.79 (-3.02, -2.56)**	0.12	1.00	2196	2636	**-14.85 (-17.97, -11.76)**	1.57	1.00	1397	2301
Biting	**0.05 (0.05, 0.05)**	0	1.00	4265	3698	**-0.57 (-0.63, -0.51)**	0.03	1.00	3120	2850
Digging	0 (0, 0.01)	0	1.00	5823	3371	**-1.5 (-1.64, -1.37)**	0.07	1.00	2315	2852
Searching substrate	0.01 (0, 0.01)	0	1.00	4842	3776	**-1.57 (-1.69, -1.44)**	0.06	1.00	2439	2503
Chewing	0.01 (0, 0.01)	0	1.00	4380	3803	**0.13 (0.1, 0.17)**	0.02	1.00	3360	2440
Grooming give	**-0.02 (-0.02, -0.02)**	0	1.00	5211	3492	**-1.96 (-2.12, -1.81)**	0.08	1.00	1707	2143
Auto grooming	0 (-0.01, 0.01)	0	1.00	6928	3594	**-1.12 (-1.23, -1.01)**	0.06	1.00	3157	2542
Handling food item	**0.04 (0.03, 0.04)**	0	1.00	6036	3770	**-0.88 (-1.01, -0.75)**	0.07	1.00	3042	2337
Picking	**0.02 (0.02, 0.02)**	0	1.00	4451	3593	**-1.24 (-1.31, -1.17)**	0.04	1.00	1717	2023
Receive grooming	-0.01 (-0.01, 0)	0	1.00	6240	2798	**-0.17 (-0.22, -0.13)**	0.02	1.00	3188	2901
Scratching	**0.03 (0.02, 0.04)**	0.01	1.00	6633	3339	0.04 (-0.13, 0.21)	0.09	1.00	3724	2461
Movement	**0.03 (0.02, 0.03)**	0	1.00	6292	3588	**-0.2 (-0.28, -0.11)**	0.04	1.00	3887	2317
Posture (Sitting)	0.06 (-0.15, 0.27)	0.11	1.00	3184	2848	**11.26 (8.78, 13.93)**	1.32	1.00	2303	2596
Posture (Standing)	0.16 (-0.06, 0.37)	0.11	1.00	3180	2987	**12.2 (9.59, 14.96)**	1.38	1.00	2186	2545
Adolescent males	-0.18 (-0.37, 0)	0.1	1.00	1503	1835	0.68 (-2.1, 3.65)	1.46	1.00	1311	1888
Adult females	-0.07 (-0.18, 0.03)	0.05	1.00	1181	1526	-0.7 (-2.54, 1.16)	0.96	1.00	811	1818
Adult females (Infant contact)	-0.02 (-0.14, 0.11)	0.06	1.00	1397	1582	-0.82 (-3.03, 1.34)	1.13	1.01	996	2149
Adult females (Infant OS)	0 (-0.24, 0.23)	0.12	1.00	3016	3042	-0.57 (-5.4, 4.1)	2.39	1.00	2111	2510
Adult males	**-0.21 (-0.35, -0.07)**	0.07	1.00	1417	1860	0.23 (-1.94, 2.48)	1.14	1.01	923	1674
Juvenile-1 females	0 (-0.14, 0.14)	0.07	1.00	1509	1948	-0.42 (-2.95, 2.04)	1.26	1.00	1267	1736
Juvenile-1 males	-0.12 (-0.26, 0.02)	0.07	1.00	1230	1799	-0.8 (-3.17, 1.48)	1.19	1.00	1117	2233
Juvenile-2 females	-0.02 (-0.14, 0.1)	0.06	1.00	1823	2620	0.3 (-1.87, 2.51)	1.12	1.00	1243	1869
Juvenile-2 males	-0.07 (-0.2, 0.05)	0.06	1.00	1107	1978	0.63 (-1.52, 2.79)	1.08	1.00	933	1826
Juvenile-3 males	**-0.18 (-0.34, -0.03)**	0.08	1.00	1451	1980	0.04 (-2.49, 2.51)	1.27	1.00	956	2034
Date										
sd(Intercept)	0.08 (0.04, 0.11)	0.02	1.00	1306	1924	0.71 (0.07, 1.35)	0.34	1.01	681	823
ID										
sd(Intercept)	0.11 (0.08, 0.15)	0.02	1.00	1403	2521	1.46 (0.89, 2.06)	0.3	1.00	1317	1509
Family										
sigma						7.96 (7.65, 8.3)	0.16	1.00	1238	2059

Table S11. Model summary results for specific behaviours (resting only) predicting the frequency and total duration of looking behaviours. Upper and lower 95% credible intervals are shown within parentheses. Bold text highlight parameter estimates where the Cis did not overlap or include zero. All R-hat (Gelman-Rubin convergence diagnostic) were less than 1.01 suggesting accurate estimates of the posterior distribution. In all cases the bulk estimated sample size (bulk_ESS) was greater than 100 times the number of chains (i.e., bulk_ESS > 400) indicating the mean was efficiently sampled in all cases.

	Frequency					Duration				
	Estimate and CIs	Est.Error	Rhat	Bulk_ESS	Tail_ESS	Estimate and CIs	Est.Error	Rhat	Bulk_ESS	Tail_ESS
Intercept	**-2.61 (-2.87, -2.38)**	0.12	1.00	1288	2186	**-85.28 (-109.1, -65.86)**	11.04	1.00	1457	1515
Rest	**-0.02 (-0.02, -0.01)**	0	1.00	4639	3414	**1.75 (1.48, 2.12)**	0.16	1.00	1644	1673
Posture (Sitting)	**0.4 (0.2, 0.61)**	0.11	1.00	2240	2371	**19.61 (7.1, 33.08)**	6.74	1.00	2227	2104
Posture (Standing)	**0.57 (0.37, 0.79)**	0.11	1.00	2215	2474	**24.84 (11.75, 39.4)**	7.06	1.00	2169	2018
Adolescent males	**-0.25 (-0.45, -0.04)**	0.1	1.00	917	1676	**14.7 (2.55, 27.05)**	6.2	1.00	1509	2577
Adult females	**-0.16 (-0.28, -0.04)**	0.06	1.01	622	1020	2.89 (-5.25, 11.11)	4.26	1.00	1020	1805
Adult females (Infant contact)	**-0.16 (-0.29, -0.02)**	0.07	1.01	776	1230	6.81 (-2.88, 16.37)	4.93	1.00	1244	2213
Adult females (Infant OS)	-0.12 (-0.37, 0.11)	0.12	1.00	1996	2283	-6.68 (-29.7, 13.79)	11.19	1.00	3622	3030
Adult males	**-0.26 (-0.42, -0.1)**	0.08	1.00	690	1429	**15.45 (6.11, 25.15)**	4.91	1.00	1103	1896
Juvenile-1 females	0.06 (-0.09, 0.21)	0.08	1.00	778	1651	-5.21 (-16.54, 6.04)	5.75	1.00	1347	1822
Juvenile-1 males	-0.04 (-0.21, 0.12)	0.08	1.01	734	1368	-0.08 (-10.75, 10.34)	5.3	1.00	1324	1690
Juvenile-2 females	-0.02 (-0.15, 0.1)	0.06	1.00	996	1792	4.98 (-4.95, 15.01)	5.1	1.00	1384	2391
Juvenile-2 males	-0.08 (-0.23, 0.06)	0.07	1.01	634	1219	**11.85 (2.74, 21.12)**	4.75	1.00	1137	1894
Juvenile-3 males	-0.14 (-0.31, 0.04)	0.09	1.00	767	1498	9.06 (-1.48, 19.7)	5.43	1.00	1293	2297
Date										
sd(Intercept)	0.14 (0.11, 0.17)	0.02	1.00	1553	2251	6.37 (3.93, 9.12)	1.33	1.00	1829	2615
ID										
sd(Intercept)	0.14 (0.11, 0.18)	0.02	1.00	1242	2128	4.98 (1.95, 7.88)	1.45	1.00	1131	1104
Family										
sigma						22.62 (20.24, 25.63)	1.36	1.00	1491	1468

Table S12. Model summary results for specific behaviours (including resting) predicting the frequency and total duration of looking behaviours. Upper and lower 95% credible intervals are shown within parentheses. Bold text highlight parameter estimates where the Cis did not overlap or include zero. All R-hat (Gelman-Rubin convergence diagnostic) were less than 1.01 suggesting accurate estimates of the posterior distribution. In all cases the bulk estimated sample size (bulk_ESS) was greater than 100 times the number of chains (i.e., bulk_ESS > 400) indicating the mean was efficiently sampled in all cases.

	Frequency					Duration				
	Estimate and CIs	Est.Error	Rhat	Bulk_ESS	Tail_ESS	Estimate and CIs	Est.Error	Rhat	Bulk_ESS	Tail_ESS
Intercept	**-2.27 (-2.68, -1.84)**	0.21	1.00	966	2006	**-23.52 (-30.41, -16.86)**	3.42	1.00	683	1345
Biting	**0.03 (0.02, 0.04)**	0.01	1.01	723	1373	**-0.28 (-0.48, -0.07)**	0.11	1.01	620	1390
Digging	-0.01 (-0.03, 0)	0.01	1.01	733	1380	**-1.2 (-1.44, -0.97)**	0.12	1.00	714	1502
Searching substrate	-0.01 (-0.02, 0)	0.01	1.01	717	1384	**-1.27 (-1.51, -1.04)**	0.12	1.01	704	1356
Chewing	-0.01 (-0.02, 0)	0.01	1.00	700	1389	**0.43 (0.23, 0.63)**	0.1	1.01	604	1295
Grooming give	**-0.04 (-0.05, -0.03)**	0.01	1.00	739	1492	**-1.66 (-1.92, -1.42)**	0.13	1.01	750	1617
Auto grooming	-0.02 (-0.03, 0)	0.01	1.00	798	1388	**-0.83 (-1.06, -0.6)**	0.12	1.01	703	1412
Handling food item	**0.02 (0.01, 0.03)**	0.01	1.00	744	1311	**-0.59 (-0.82, -0.35)**	0.12	1.00	740	1622
Picking	0 (-0.01, 0.01)	0.01	1.01	686	1346	**-0.95 (-1.15, -0.74)**	0.11	1.01	614	1380
Receive grooming	**-0.03 (-0.04, -0.01)**	0.01	1.00	763	1644	0.12 (-0.08, 0.32)	0.1	1.01	589	1313
Resting	**-0.02 (-0.03, -0.01)**	0.01	1.00	723	1455	**0.3 (0.1, 0.51)**	0.1	1.01	594	1392
Scratching	0.01 (0, 0.03)	0.01	1.00	999	2143	**0.34 (0.07, 0.61)**	0.13	1.00	849	1502
Movement	0.01 (0, 0.02)	0.01	1.00	761	1452	0.09 (-0.11, 0.31)	0.11	1.01	660	1390
Posture (Sitting)	0.08 (-0.12, 0.29)	0.11	1.00	4981	3293	**11.08 (8.55, 13.68)**	1.3	1.00	3032	2775
Posture (Standing)	0.17 (-0.04, 0.38)	0.11	1.00	5030	2911	**12.22 (9.55, 14.9)**	1.36	1.00	3023	2656
Adolescent males	-0.18 (-0.37, 0)	0.09	1.00	2795	3024	0.71 (-2.14, 3.62)	1.49	1.00	2042	2563
Adult females	-0.07 (-0.18, 0.03)	0.05	1.00	1518	2238	-0.68 (-2.56, 1.21)	0.94	1.00	1208	1960
Adult females (Infant contact)	-0.02 (-0.14, 0.11)	0.06	1.00	1851	2666	-0.81 (-2.99, 1.35)	1.12	1.00	1332	2204
Adult females (Infant OS)	-0.01 (-0.25, 0.21)	0.12	1.00	4098	2891	-0.26 (-4.87, 4.38)	2.36	1.00	3575	2639
Adult males	**-0.21 (-0.35, -0.07)**	0.07	1.00	1846	2583	0.3 (-1.89, 2.46)	1.1	1.00	1464	1992
Juvenile-1 females	0 (-0.14, 0.14)	0.07	1.00	2110	2542	-0.37 (-2.93, 2.09)	1.25	1.00	1720	2144
Juvenile-1 males	-0.12 (-0.27, 0.02)	0.07	1.00	2057	2667	-0.55 (-2.92, 1.9)	1.23	1.00	1376	1957
Juvenile-2 females	-0.02 (-0.14, 0.1)	0.06	1.00	2542	2894	0.41 (-1.8, 2.64)	1.14	1.00	1937	2618
Juvenile-2 males	-0.08 (-0.2, 0.05)	0.07	1.00	1782	2583	0.75 (-1.33, 2.88)	1.07	1.00	1173	1722
Juvenile-3 males	**-0.18 (-0.33, -0.03)**	0.08	1.00	1923	2824	0.05 (-2.43, 2.55)	1.25	1.00	1634	2636
Date						Date				
sd(Intercept)	0.08 (0.04, 0.11)	0.02	1.01	1369	1760	0.7 (0.04, 1.34)	0.34	1.00	739	703
ID						ID				
sd(Intercept)	0.11 (0.08, 0.15)	0.02	1.00	1758	2939	1.45 (0.9, 2.05)	0.3	1.00	1550	2233
Family						Family				
sigma						7.95 (7.64, 8.27)	0.16	1.00	3147	2615

Reactionary models

Table S13. Model summary results for all reactionary models with the frequency response variable, displaying parameter estimates and credible intervals. In all cases the bulk effective sample size was greater than 100 times the number of chains (i.e., Bulk ESS > 400) indicating efficient sampling of the mean of the distributions. The Gelman-Rubin convergence diagnostic was less than 1.01 in all cases suggesting accurate estimates of the posterior distributions. Bold text highlights where credible intervals did not overlap or include zero. None of these models shared considerable weight when stacked. IE refers to inter-species (or heterospecific) encounters. WE refers to within-species encounters (i.e., foreign males or other groups).

	Aggression	Mating	Female calls	Male calls	Passive IE	Active IE	Alarm	Dog encounter	WE
Intercept	**-2.24 (-2.43, -2.04)**	**-2.16 (-2.31, -2.01)**	**-1.88 (-2.02, -1.73)**	**-2.36 (-2.57, -2.15)**	**-2.09 (-2.27, -1.91)**	**-2.38 (-2.75, -2.02)**	**-1.96 (-2.2, -1.72)**	**-1.75 (-2, -1.5)**	**-2.35 (-2.59, -2.12)**
No event	**-0.27 (-0.39, -0.15)**	**0.06 (-0.06, 0.18)**	**-0.26 (-0.37, -0.16)**	-0.08 (-0.22, 0.05)	-0.05 (-0.2, 0.11)	0.08 (-0.22, 0.38)	-0.13 (-0.32, 0.06)	**-0.31 (-0.51, -0.11)**	0.11 (-0.05, 0.28)
Event ongoing	0 (-0.11, 0.12)	0.08 (-0.18, 0.33)	**-0.2 (-0.35, -0.04)**	-0.12 (-0.3, 0.06)	0.01 (-0.16, 0.18)	0.22 (-0.2, 0.63)	-0.17 (-0.5, 0.15)	-0.13 (-0.46, 0.19)	-0.07 (-0.25, 0.1)
5 - 10 mins post event	-0.08 (-0.16, 0.01)	-0.06 (-0.22, 0.09)	-0.15 (-0.3, 0)	0.08 (-0.04, 0.21)	0.21 (0.03, 0.4)	0.18 (-0.21, 0.57)	-0.01 (-0.26, 0.24)	-0.06 (-0.35, 0.23)	0.15 (-0.06, 0.37)
10 - 15 mins post event	**-0.14 (-0.23, -0.05)**	0.06 (-0.1, 0.21)	-0.08 (-0.23, 0.06)	0 (-0.13, 0.14)	0.21 (0, 0.42)	-0.46 (-1, 0.07)	-0.1 (-0.37, 0.18)	-0.19 (-0.51, 0.14)	0.05 (-0.17, 0.26)
15 + mins post event	**-0.08 (-0.15, -0.02)**	0.09 (-0.01, 0.2)	**-0.23 (-0.32, -0.13)**	0.05 (-0.05, 0.15)	0.03 (-0.12, 0.18)	0.02 (-0.27, 0.33)	-0.07 (-0.25, 0.13)	**-0.3 (-0.5, -0.1)**	0.14 (-0.01, 0.3)
Rank	0 (0, 0.01)			0 (0, 0.01)		0 (0, 0.01)			0 (0, 0.01)
Visibility	0 (0, 0)			0 (0, 0)		0 (0, 0)	0 (0, 0)	0 (0, 0)	0 (0, 0)
Tolerance									
Spatial position (Peripheral)							**0.05 (0.01, 0.09)**	0.05 (0, 0.09)	0.05 (0, 0.09)
Number of neighbours (5m)							**-0.04 (-0.05, -0.03)**	**-0.04 (-0.06, -0.03)**	**-0.04 (-0.05, -0.03)**
Time spent engaged	**-0.01 (-0.01, -0.01)**	**-0.01 (-0.01, -0.01)**	**-0.01 (-0.01, -0.01)**	**-0.01 (-0.01, -0.01)**	**-0.01 (-0.01, -0.01)**	**-0.01 (-0.01, -0.01)**	**-0.01 (-0.01, -0.01)**	**-0.01 (-0.01, -0.01)**	**-0.01 (-0.01, -0.01)**
Adolescent males	**-0.29 (-0.51, -0.08)**	**-0.47 (-0.66, -0.26)**	**-0.45 (-0.64, -0.25)**	**-0.29 (-0.51, -0.07)**	**-0.46 (-0.66, -0.26)**	**-0.3 (-0.53, -0.08)**	**-0.44 (-0.64, -0.25)**	**-0.43 (-0.63, -0.23)**	**-0.32 (-0.54, -0.09)**
Adult females	**-0.15 (-0.26, -0.03)**	**-0.19 (-0.3, -0.07)**	**-0.19 (-0.3, -0.07)**	**-0.16 (-0.27, -0.04)**	**-0.19 (-0.31, -0.08)**	**-0.16 (-0.28, -0.05)**	**-0.19 (-0.3, -0.08)**	**-0.19 (-0.31, -0.08)**	**-0.17 (-0.28, -0.06)**
Adult females (Infant contact)	**-0.18 (-0.31, -0.05)**	**-0.22 (-0.35, -0.08)**	**-0.21 (-0.34, -0.08)**	**-0.19 (-0.32, -0.06)**	**-0.22 (-0.34, -0.09)**	**-0.19 (-0.32, -0.07)**	**-0.18 (-0.31, -0.05)**	**-0.19 (-0.32, -0.06)**	**-0.17 (-0.29, -0.04)**
Adult females (Infant OS)	**-0.05 (-0.29, 0.18)**	-0.08 (-0.32, 0.15)	-0.07 (-0.29, 0.15)	-0.05 (-0.28, 0.19)	-0.08 (-0.32, 0.16)	-0.05 (-0.29, 0.18)	-0.07 (-0.31, 0.15)	-0.07 (-0.31, 0.15)	-0.06 (-0.3, 0.16)
Adult males	**-0.24 (-0.43, -0.06)**	**-0.43 (-0.58, -0.28)**	**-0.42 (-0.57, -0.28)**	**-0.25 (-0.43, -0.06)**	**-0.43 (-0.58, -0.28)**	**-0.25 (-0.44, -0.07)**	**-0.41 (-0.56, -0.26)**	**-0.41 (-0.56, -0.26)**	**-0.26 (-0.44, -0.08)**
Juvenile-1 females	**0.07 (-0.07, 0.21)**	0.07 (-0.08, 0.21)	0.07 (-0.08, 0.22)	0.06 (-0.08, 0.21)	0.06 (-0.08, 0.21)	0.06 (-0.09, 0.2)	0.08 (-0.06, 0.23)	0.08 (-0.07, 0.22)	0.08 (-0.07, 0.22)
Juvenile-1 males	**-0.01 (-0.17, 0.14)**	-0.08 (-0.24, 0.07)	-0.07 (-0.23, 0.08)	-0.02 (-0.17, 0.13)	-0.08 (-0.24, 0.06)	-0.02 (-0.18, 0.13)	-0.03 (-0.19, 0.12)	-0.04 (-0.19, 0.12)	0 (-0.14, 0.16)
Juvenile-2 females	**-0.06 (-0.18, 0.06)**	-0.06 (-0.19, 0.06)	-0.06 (-0.18, 0.07)	-0.06 (-0.19, 0.06)	-0.07 (-0.19, 0.05)	-0.07 (-0.19, 0.06)	-0.05 (-0.17, 0.08)	-0.05 (-0.17, 0.07)	-0.06 (-0.18, 0.06)
Juvenile-2 males	**-0.06 (-0.2, 0.07)**	-0.13 (-0.26, 0.01)	-0.12 (-0.26, 0.02)	-0.07 (-0.21, 0.07)	-0.13 (-0.27, 0)	-0.08 (-0.22, 0.06)	-0.11 (-0.24, 0.03)	-0.11 (-0.25, 0.02)	-0.06 (-0.2, 0.08)
Juvenile-3 males	**-0.11 (-0.28, 0.07)**	**-0.25 (-0.41, -0.08)**	**-0.23 (-0.4, -0.06)**	-0.11 (-0.28, 0.08)	**-0.25 (-0.41, -0.08)**	-0.12 (-0.3, 0.06)	**-0.22 (-0.38, -0.07)**	**-0.21 (-0.38, -0.05)**	-0.11 (-0.28, 0.07)
Date									
sd(Intercept)	0.16 (0.13, 0.2)	0.17 (0.13, 0.21)	0.16 (0.13, 0.2)	0.16 (0.13, 0.2)	0.17 (0.13, 0.2)	0.17 (0.14, 0.21)	0.15 (0.12, 0.19)	0.16 (0.13, 0.2)	0.16 (0.13, 0.2)
ID									
sd(Intercept)	0.12 (0.1, 0.16)	0.13 (0.1, 0.17)	0.13 (0.1, 0.17)	0.13 (0.1, 0.17)	0.13 (0.1, 0.17)	0.12 (0.09, 0.16)	0.13 (0.1, 0.16)	0.13 (0.1, 0.17)	0.12 (0.09, 0.16)

Table S14. Model summary results for all reactionary models with the duration response variable, displaying parameter estimates and credible intervals. In all cases the bulk effective sample size was greater than 100 times the number of chains (i.e., Bulk ESS > 400) indicating efficient sampling of the mean of the distributions. The Gelman-Rubin convergence diagnostic was less than 1.01 in all cases suggesting accurate estimates of the posterior distributions. Bold text highlights where credible intervals did not overlap or include zero. Only time since wahoo and within-species encounter models had considerable weight when stacked. IE refers to inter-species (or heterospecific) encounters. WE refers to within-species encounters (i.e., foreign males or other groups).

	Aggression	Mating	Female calls	Male calls	Passive IE	Active IE	Alarm	DE	WE
Intercept	**-70.31 (-76.41, -64.97)**	**-59.1 (-64.14, -54.43)**	**-61.72 (-67, -56.89)**	**-67.06 (-73.17, -61.69)**	**-64.28 (-70.24, -58.59)**	**-81.84 (-91.64, -72.22)**	**-68.17 (-75.3, -61.68)**	**-72.47 (-80.16, -65.29)**	**-68.46 (-75.14, -62.37)**
No event	1.99 (-0.7, 4.56)	-2.02 (-4.69, 0.66)	0.91 (-2.12, 3.91)	-0.08 (-3.13, 2.93)	3.86 (-0.12, 8.03)	**11.18 (3.7, 19.42)**	-1.75 (-6.21, 2.96)	1.98 (-3.3, 7.57)	0.43 (-3.24, 4.2)
Event ongoing	**6.94 (4.02, 9.68)**	-1.03 (-7.32, 5.15)	1.58 (-2.59, 5.66)	**6.47 (2.49, 10.4)**	0.17 (-4.15, 4.65)	**13.59 (2.19, 25.06)**	**7.94 (0.6, 15.43)**	4.3 (-3.99, 12.55)	**5.32 (1.44, 9.38)**
5 - 10 mins post event	1.01 (-1.18, 3.16)	-1.24 (-4.78, 2.34)	2.44 (-1.82, 6.57)	-1.56 (-4.67, 1.6)	1.36 (-4.07, 6.77)	**10.74 (1.02, 20.77)**	-1.6 (-7.81, 4.91)	-2.47 (-10.96, 5.65)	-3.8 (-8.93, 1.39)
10 - 15 mins post event	-0.32 (-2.59, 2.01)	-2.66 (-6.24, 0.93)	1.81 (-2.45, 5.88)	-2.45 (-5.75, 0.9)	1.7 (-4.1, 7.57)	**19.98 (9.21, 31.42)**	1.88 (-4.68, 8.38)	-0.49 (-9.06, 8)	-2.65 (-8.13, 2.69)
15 + mins post event	0.32 (-1.27, 1.9)	-2.4 (-4.97, 0.12)	0.09 (-2.68, 2.93)	**-2.94 (-5.21, -0.61)**	2.67 (-1.33, 6.74)	**12.62 (4.94, 20.81)**	-2.23 (-6.74, 2.51)	0.98 (-4.36, 6.67)	-0.58 (-4.23, 3.14)
Rank	-0.01 (-0.05, 0.02)			-0.01 (-0.05, 0.03)		-0.01 (-0.04, 0.03)			-0.01 (-0.05, 0.03)
Visibility	**0.14 (0.11, 0.18)**			**0.14 (0.11, 0.17)**		**0.14 (0.11, 0.17)**	**0.14 (0.1, 0.17)**	**0.14 (0.11, 0.18)**	**0.13 (0.1, 0.16)**
Tolerance									
Spatial position (Peripheral)							-0.16 (-1.19, 0.84)	-0.15 (-1.2, 0.9)	-0.29 (-1.33, 0.72)
Number of neighbours (5m)							-0.1 (-0.38, 0.17)	-0.1 (-0.36, 0.17)	-0.17 (-0.45, 0.1)
Time spent not engaged	**1.73 (1.62, 1.85)**	**1.78 (1.67, 1.91)**	**1.78 (1.66, 1.91)**	**1.72 (1.61, 1.84)**	**1.78 (1.66, 1.91)**	**1.74 (1.63, 1.86)**	**1.74 (1.63, 1.86)**	**1.75 (1.63, 1.87)**	**1.72 (1.61, 1.84)**
Adolescent males	1.82 (-2.21, 5.79)	2.92 (-0.67, 6.64)	3.02 (-0.75, 6.78)	1.92 (-2.08, 5.88)	3.1 (-0.52, 6.8)	2.31 (-1.38, 6.32)	2.55 (-0.94, 6.21)	2.53 (-1.09, 6.11)	2.18 (-1.71, 6.14)
Adult females	0.25 (-2.08, 2.59)	0.62 (-1.72, 3.04)	0.65 (-1.82, 3.09)	0.41 (-1.97, 2.88)	0.79 (-1.61, 3.27)	0.57 (-1.74, 2.88)	0.55 (-1.78, 2.93)	0.47 (-1.86, 2.79)	0.4 (-1.94, 2.73)
Adult females (Infant contact)	1.35 (-1.46, 4.08)	1.64 (-1.21, 4.57)	1.62 (-1.35, 4.52)	1.43 (-1.3, 4.25)	1.85 (-0.93, 4.66)	1.68 (-1.09, 4.44)	1.68 (-1.13, 4.39)	1.6 (-1.05, 4.3)	1.47 (-1.17, 4.24)
Adult females (Infant OS)	1.69 (-5.04, 7.95)	2.41 (-4.05, 8.91)	2.33 (-4.31, 9.06)	1.66 (-4.34, 7.93)	2.81 (-3.78, 9.18)	2.24 (-4.31, 8.2)	2.29 (-3.88, 8.33)	2.25 (-4.07, 8.62)	2.22 (-4.2, 8.53)
Adult males	2.11 (-1.09, 5.33)	**3.43 (0.75, 6.19)**	**3.5 (0.81, 6.17)**	2.2 (-1.1, 5.4)	**3.56 (0.8, 6.32)**	2.51 (-0.59, 5.61)	**2.87 (0.28, 5.41)**	**2.79 (0.11, 5.42)**	2.2 (-0.94, 5.4)
Juvenile-1 females	-2.28 (-5.35, 0.77)	-2.21 (-5.42, 0.97)	-2.24 (-5.47, 0.92)	-2.06 (-5.27, 0.99)	-2.11 (-5.4, 1.02)	-2.17 (-5.25, 0.88)	-2.19 (-5.39, 0.87)	-2.17 (-5.4, 0.91)	-2.04 (-5.15, 0.94)
Juvenile-1 males	-2.19 (-5.21, 0.79)	-1.95 (-4.95, 1.04)	-1.96 (-5.02, 1.16)	-1.89 (-4.93, 1.15)	-1.64 (-4.67, 1.42)	-2 (-4.91, 0.94)	-1.77 (-4.77, 1.2)	-1.78 (-4.64, 1.07)	-1.7 (-4.71, 1.27)
Juvenile-2 females	-0.57 (-3.26, 2.27)	-0.18 (-2.96, 2.78)	-0.14 (-3.12, 2.78)	-0.17 (-3.09, 2.78)	-0.05 (-2.84, 2.84)	-0.28 (-3.15, 2.57)	-0.41 (-3.21, 2.55)	-0.46 (-3.35, 2.35)	-0.31 (-3.14, 2.46)
Juvenile-2 males	2.13 (-0.57, 4.75)	**2.99 (0.39, 5.7)**	**3.03 (0.28, 5.75)**	2.33 (-0.37, 5.01)	**3.18 (0.48, 5.83)**	**2.65 (0.05, 5.32)**	2.55 (-0.05, 5.14)	2.54 (-0.03, 5.1)	2.15 (-0.4, 4.78)
Juvenile-3 males	1.24 (-2.08, 4.7)	2.18 (-0.85, 5.31)	2.28 (-0.95, 5.41)	1.33 (-2.19, 4.69)	2.34 (-0.8, 5.4)	1.55 (-1.65, 4.69)	1.7 (-1.38, 4.82)	1.72 (-1.27, 4.65)	1.14 (-2.08, 4.37)
Date									
sd(Intercept)	2.07 (1.35, 2.85)	2.14 (1.4, 2.94)	2.17 (1.41, 2.97)	2 (1.28, 2.75)	2.16 (1.45, 2.91)	2.11 (1.39, 2.91)	2.04 (1.31, 2.82)	2.02 (1.3, 2.75)	1.95 (1.23, 2.7)
ID									
sd(Intercept)	1.23 (0.24, 2.11)	1.28 (0.33, 2.13)	1.28 (0.15, 2.19)	1.21 (0.2, 2.08)	1.28 (0.22, 2.2)	1.16 (0.13, 2.05)	1.21 (0.17, 2.08)	1.13 (0.15, 2.02)	1.09 (0.1, 1.98)
Family									
sigma	10.34 (9.88, 10.87)	10.57 (10.09, 11.11)	10.56 (10.08, 11.08)	10.3 (9.83, 10.79)	10.56 (10.07, 11.08)	10.36 (9.89, 10.87)	10.36 (9.89, 10.88)	10.42 (9.93, 10.92)	10.31 (9.85, 10.8)

Table S15. Summary results for model exploring the relationship between the frequency of looking bouts and the number of social threats within 5 meters. Also included are dominance rank of the focal animal, number of neighbours within 5 meters, and visibility as these factors could influence the aforementioned relationship. The remainder of the population-level and group-level factors are the same as the minimal model.

	Estimate	Est.Error	l-95% CI	u-95% CI	Rhat	Bulk_ESS	Tail_ESS
Intercept	-2.14	0.1	-2.32	-1.95	1.00	1418	2279
Count of social threats in 5 meters	0.08	0.01	0.05	0.1	1.00	3177	2930
Time spent engaged	-0.01	0	-0.01	-0.01	1.00	4029	3149
Dominance rank	0	0	0	0	1.00	1804	2753
Number of neighbours within 5 meters	-0.08	0.01	-0.09	-0.06	1.00	3179	3211
Visibility	0	0	0	0	1.00	4248	2811
Adolescent males	-0.32	0.11	-0.53	-0.1	1.00	1542	2139
Adult females	-0.17	0.06	-0.28	-0.05	1.00	1175	1980
Adult females (Infant contact)	-0.17	0.07	-0.3	-0.04	1.00	1322	2318
Adult females (Infant OS)	-0.06	0.12	-0.29	0.18	1.00	2555	2912
Adult males	-0.26	0.09	-0.44	-0.07	1.00	1188	1815
Juvenile-1 females	0.08	0.07	-0.07	0.22	1.00	1462	2263
Juvenile-1 males	0	0.08	-0.15	0.16	1.00	1123	2006
Juvenile-2 females	-0.06	0.06	-0.18	0.06	1.00	1726	2376
Juvenile-2 males	-0.08	0.07	-0.21	0.06	1.00	1085	2128
Juvenile-3 males	-0.13	0.09	-0.31	0.05	1.00	1270	2066
Date							
sd(Intercept)	0.16	0.02	0.13	0.2	1.00	1496	2178
ID							
sd(Intercept)	0.12	0.02	0.09	0.16	1.00	1735	2752

Table S16. Summary results for model exploring the relationship between the total duration of looking bouts and the number of social threats within 5 meters. Also included are dominance rank of the focal animal, number of neighbours within 5 meters, and visibility as these factors could influence the aforementioned relationship. The remainder of the population-level and group-level factors are the same as the minimal model.

	Estimate	Est.Error	l-95% CI	u-95% CI	Rhat	Bulk_ESS	Tail_ESS
Intercept	-68.34	2.72	-73.63	-63.24	1.00	1609	2364
Count of social threats in 5 meters	1.27	0.32	0.63	1.91	1.00	3084	3308
Number of neighbours within 5 meters	-0.55	0.17	-0.89	-0.22	1.00	3346	3086
Dominance rank	-0.04	0.02	-0.08	0	1.00	2231	2412
Visibility	0.14	0.02	0.11	0.17	1.00	4878	3252
Time spent not engaged	1.73	0.06	1.62	1.85	1.00	2063	2581
Adolescent males	1.66	2.04	-2.33	5.64	1.00	1423	2074
Adult females	0.14	1.22	-2.31	2.48	1.00	1139	2082
Adult females (Infant contact)	1.17	1.43	-1.74	3.96	1.00	1421	2318
Adult females (Infant OS)	2.35	3.21	-3.99	8.64	1.00	3487	2875
Adult males	1.95	1.69	-1.36	5.28	1.00	1099	1695
Juvenile-1 females	-2.33	1.59	-5.49	0.74	1.00	1791	2489
Juvenile-1 males	-2.22	1.57	-5.28	0.9	1.00	1547	2470
Juvenile-2 females	-0.58	1.44	-3.44	2.19	1.00	1791	3070
Juvenile-2 males	1.98	1.39	-0.76	4.67	1.00	1291	2272
Juvenile-3 males	0.82	1.72	-2.6	4.07	1.00	1154	2096
Date							
sd(Intercept)	2.01	0.38	1.3	2.8	1.00	1458	1998
ID							
sd(Intercept)	1.3	0.45	0.34	2.12	1.00	999	954
Family							
sigma	10.33	0.25	9.87	10.84	1.00	1838	2639

Table S17. Summary results for model exploring the relationship between the frequency of looking bouts and the interaction between probability of occurrence of leopards and several other reported risk factors, including time spent engaged, number of neighbours, spatial position. Also included are dominance rank of the focal animal, and visibility as these factors could influence the aforementioned relationship. The remainder of the population-level and group-level factors are the same as the minimal model.

	Estimate	Est.Error	l-95% CI	u-95% CI	Rhat	Bulk_ESS	Tail_ESS
Intercept	-1.97	0.15	-2.26	-1.69	1.00	1902	2485
Leopard risk	-0.38	0.17	-0.7	-0.06	1.00	2355	2794
Number of neighbours (5m)	-0.17	0.03	-0.23	-0.11	1.00	2741	2860
Spatial position (Peripheral)	-0.23	0.11	-0.45	-0.01	1.00	2789	2859
Time spent engaged	-0.01	0	-0.02	0	1.00	3778	2684
Visibility	0	0	0	0	1.00	4524	3213
Rank	0	0	0	0.01	1.00	2154	2467
Adolescent males	-0.31	0.11	-0.53	-0.1	1.01	1344	2025
Adult females	-0.17	0.06	-0.28	-0.05	1.00	1181	2178
Adult females (Infant contact)	-0.16	0.07	-0.29	-0.04	1.00	1492	2526
Adult females (Infant OS)	-0.06	0.12	-0.3	0.17	1.00	2974	2801
Adult males	-0.27	0.09	-0.44	-0.08	1.00	1277	2005
Juvenile-1 females	0.08	0.07	-0.06	0.22	1.00	1693	2311
Juvenile-1 males	0.01	0.08	-0.14	0.16	1.00	1262	2255
Juvenile-2 females	-0.06	0.06	-0.18	0.06	1.00	1901	2938
Juvenile-2 males	-0.07	0.07	-0.21	0.07	1.00	1080	1675
Juvenile-3 males	-0.11	0.09	-0.28	0.07	1.00	1226	1869
Leopard risk : Number of neighbours	0.19	0.05	0.1	0.29	1.00	2734	2747
Leopard risk : Spatial position (Peripheral)	0.41	0.17	0.08	0.74	1.00	2800	2739
Leopard risk : Time spent engaged	0	0.01	-0.02	0.01	1.00	3763	2741
Date							
sd(Intercept)	0.16	0.02	0.13	0.2	1.00	1327	1953
ID							
sd(Intercept)	0.12	0.02	0.09	0.16	1.00	1748	2503

Table S18. Summary results for model exploring the relationship between the total duration of looking bouts and the interaction between probability of occurrence of leopards and several other reported risk factors, including time spent not engaged, number of neighbours, spatial position. Also included are dominance rank of the focal animal, and visibility as these factors could influence the aforementioned relationship. The remainder of the population-level and group-level factors are the same as the minimal model.

	Estimate	Est.Error	l-95% CI	u-95% CI	Rhat	Bulk_ESS	Tail_ESS
Intercept	-60.58	5.83	-71.91	-49.17	1.00	3458	3325
Leopard risk	-13.99	7.73	-29.64	0.99	1.00	3527	2937
Number of neighbours (5m)	-1.29	0.73	-2.69	0.14	1.00	5192	3446
Spatial position (Peripheral)	1.96	2.68	-3.35	7.19	1.00	5272	3199
Time spent not engaged	1.33	0.19	0.96	1.7	1.00	3783	3054
Visibility	0.14	0.02	0.1	0.17	1.00	8330	3240
Rank	-0.01	0.02	-0.05	0.03	1.00	4045	3121
Adolescent males	1.98	2.05	-2.03	5.94	1.00	2363	2830
Adult females	0.4	1.21	-1.93	2.79	1.00	1707	2416
Adult females (Infant contact)	1.47	1.43	-1.27	4.24	1.00	1950	3102
Adult females (Infant OS)	2.25	3.29	-4.35	8.62	1.00	5582	3011
Adult males	2.42	1.67	-0.95	5.58	1.00	1843	2509
Juvenile-1 females	-2.02	1.55	-5.09	0.99	1.00	2351	2828
Juvenile-1 males	-1.85	1.55	-4.85	1.13	1.00	2307	2915
Juvenile-2 females	-0.29	1.45	-3.08	2.55	1.00	2432	3181
Juvenile-2 males	2.44	1.38	-0.21	5.18	1.00	1875	2519
Juvenile-3 males	1.46	1.74	-2.11	4.75	1.00	1869	2809
Leopard risk : Number of neighbours	1.83	1.11	-0.3	3.99	1.00	4950	3317
Leopard risk : Spatial position (Peripheral)	-3.02	4	-10.8	4.74	1.00	5042	3022
Leopard risk : Time spent not engaged	0.62	0.27	0.1	1.16	1.00	3748	2904
Date							
sd(Intercept)	2.04	0.37	1.36	2.84	1.00	1680	2559
ID							
sd(Intercept)	1.17	0.46	0.18	2.04	1.00	1174	1220
Family							
sigma	10.38	0.25	9.9	10.9	1.00	4636	2590

Table S19. Summary results for model exploring the relationship between the frequency of looking bouts and the interaction between habitat type and several other reported risk factors, including time spent engaged, number of neighbours, spatial position. Also included are dominance rank of the focal animal, and visibility as these factors could influence the aforementioned relationship. The remainder of the population-level and group-level factors are the same as the minimal model.

	Estimate	Est.Error	l-95% CI	u-95% CI	Rhat	Bulk_ESS	Tail_ESS
Intercept	-2.25	0.13	-2.52	-2	1.00	928	1611
Camp	-0.02	0.12	-0.27	0.22	1.00	893	1564
Farm	0.04	0.16	-0.26	0.35	1.00	978	1733
Forest	-0.41	0.13	-0.69	-0.16	1.00	901	1903
Grassland	0.13	0.1	-0.08	0.33	1.00	623	1232
Road	-0.03	0.12	-0.28	0.21	1.00	722	1640
Rock	-0.33	0.15	-0.61	-0.03	1.00	945	2037
Woodland	0.07	0.11	-0.14	0.28	1.00	708	1376
Spatial position (Peripheral)	0.12	0.1	-0.08	0.31	1.00	789	1257
Number of neighbours (5m)	-0.01	0.03	-0.07	0.06	1.00	734	1222
Time spent engaged	-0.01	0	-0.02	-0.01	1.00	1391	2139
Rank	0	0	0	0.01	1.00	1862	2406
Visibility	0	0	0	0	1.00	4307	3030
Adolescent males	-0.3	0.11	-0.51	-0.07	1.01	1519	2739
Adult females	-0.15	0.06	-0.26	-0.03	1.01	1029	1501
Adult females (Infant contact)	-0.16	0.07	-0.29	-0.03	1.01	1268	1903
Adult females (Infant OS)	-0.07	0.12	-0.32	0.17	1.00	2553	2396
Adult males	-0.25	0.09	-0.43	-0.07	1.01	1121	2241
Juvenile-1 females	0.08	0.07	-0.06	0.22	1.00	1594	2116
Juvenile-1 males	0.01	0.08	-0.14	0.17	1.00	1007	1759
Juvenile-2 females	-0.03	0.06	-0.16	0.08	1.00	1834	2244
Juvenile-2 males	-0.07	0.07	-0.2	0.07	1.01	1136	1562
Juvenile-3 males	-0.12	0.09	-0.29	0.06	1.01	1009	2058
Camp : Peripheral	-0.04	0.12	-0.27	0.18	1.00	1048	1633
Farm : Peripheral	0.11	0.14	-0.19	0.39	1.00	1094	1928
Forest : Peripheral	-0.17	0.14	-0.45	0.12	1.00	1283	2029
Grassland : Peripheral	-0.09	0.1	-0.29	0.11	1.00	828	1452
Road : Peripheral	-0.13	0.12	-0.37	0.11	1.00	1025	1541
Rock : Peripheral	-0.34	0.15	-0.62	-0.05	1.00	1298	1879
Woodland : Peripheral	-0.08	0.11	-0.28	0.13	1.00	914	1265
Camp : Number of neighbours	-0.01	0.04	-0.09	0.07	1.00	890	1707
Farm : Number of neighbours	-0.01	0.05	-0.11	0.08	1.00	1152	1973
Forest : Number of neighbours	-0.02	0.04	-0.1	0.07	1.00	1044	1887
Grassland : Number of neighbours	-0.05	0.03	-0.11	0.02	1.00	747	1496
Road : Number of neighbours	-0.02	0.04	-0.1	0.06	1.00	838	1805
Rock : Number of neighbours	-0.03	0.04	-0.11	0.05	1.00	938	1914
Woodland : Number of neighbours	-0.02	0.04	-0.09	0.05	1.00	793	1530
Camp : Time engaged	0.01	0	0	0.02	1.00	1756	2659
Farm : Time engaged	0.01	0.01	0	0.02	1.00	2143	3135
Forest : Time engaged	0.01	0.01	0	0.02	1.00	1868	2585
Grassland : Time engaged	0	0	-0.01	0.01	1.00	1497	2248

Road : Time engaged	0.01	0.01	0	0.02	1.00	1716	2392
Rock : Time engaged	0	0.01	-0.01	0.01	1.00	1988	2348
Woodland : Time engaged	0	0	-0.01	0.01	1.00	1475	1975
Date							
sd(Intercept)	0.16	0.02	0.12	0.19	1.00	1284	2328
ID							
sd(Intercept)	0.12	0.02	0.09	0.15	1.00	1485	2316

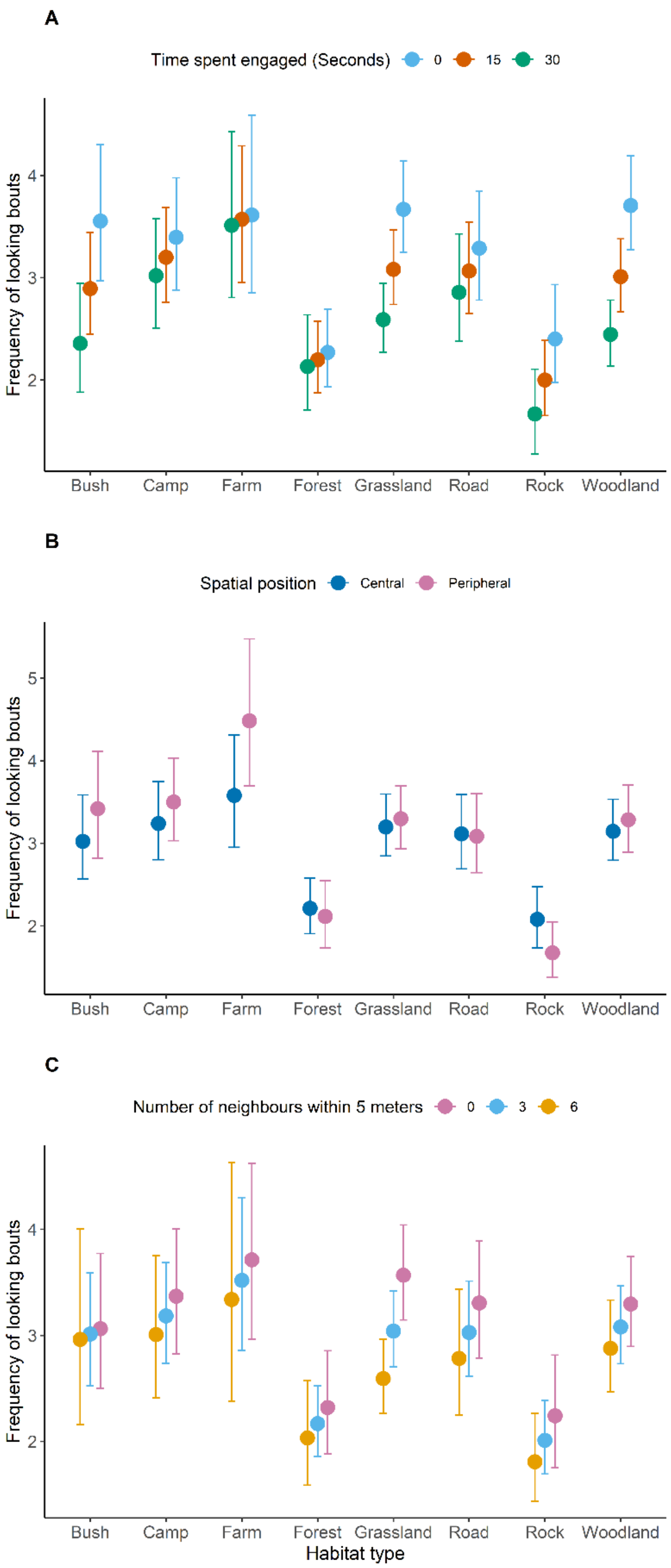

Figure S1. Conditional effects plots showing how the frequency of looking bouts varied according to the interaction between habitat type and (panel A) time spent engaged, (B) spatial position, and (C) number of neighbours within 5 meters. For time spent engaged the conditional means and credible intervals (2.5% and 97.5% quantiles) are shown for specific values of time spent engaged (i.e., 0, 15, 30 seconds) for graphical purposes only. This was also done for number of neighbours at different values (0, 3, and 6 neighbours).

Table S20. Summary results for model exploring the relationship between the total duration of looking bouts and the interaction between habitat type and several other reported risk factors, including time spent not engaged, number of neighbours, spatial position. Also included are dominance rank of the focal animal, and visibility as these factors could influence the aforementioned relationship. The remainder of the population-level and group-level factors are the same as the minimal model.

	Estimate	Est.Error	l-95% CI	u-95% CI	Rhat	Bulk_ESS	Tail_ESS
Intercept	-63.47	5.06	-73.63	-53.8	1.00	556	1355
Camp	-4.8	5.53	-15.42	6.31	1.00	596	1085
Farm	0.72	6.36	-11.77	13.02	1.00	715	1527
Forest	-12.62	6.31	-25.4	-0.34	1.00	706	1442
Grassland	-3.68	4.68	-12.63	5.57	1.01	474	930
Road	0.88	5.37	-9.69	11.23	1.00	596	1215
Rock	-6.09	6.16	-18.11	6.1	1.00	718	1369
Woodland	-9.53	4.77	-18.74	-0.12	1.00	498	1032
Spatial position (Peripheral)	-1.1	2.7	-6.62	4.16	1.00	451	1011
Number of neighbours (5m)	-1.34	0.9	-3.12	0.4	1.00	581	1267
Time spent not engaged	1.63	0.16	1.33	1.94	1.01	607	1072
Rank	-0.01	0.02	-0.05	0.03	1.00	2368	2382
Visibility	0.13	0.02	0.09	0.16	1.00	6475	3013
Adolescent males	2.25	2.04	-1.7	6.34	1.00	1291	2253
Adult females	0.41	1.23	-2.09	2.76	1.00	827	1958
Adult females (Infant contact)	1.62	1.43	-1.25	4.38	1.00	1034	2253
Adult females (Infant OS)	1.68	3.32	-4.8	8.17	1.00	3438	2495
Adult males	2.53	1.66	-0.8	5.72	1.00	1054	1926
Juvenile-1 females	-1.98	1.59	-5.13	1.11	1.00	1375	2031
Juvenile-1 males	-2.02	1.53	-5.03	0.86	1.00	1236	2315
Juvenile-2 females	-0.71	1.47	-3.59	2.08	1.00	1301	2472
Juvenile-2 males	2.34	1.39	-0.48	5.06	1.00	965	1908
Juvenile-3 males	1.89	1.72	-1.46	5.23	1.00	1058	2242
Camp : Peripheral	2.39	3.3	-3.99	8.95	1.00	572	1308
Farm : Peripheral	1.73	4.12	-6.38	9.68	1.00	1052	1852
Forest : Peripheral	2.06	3.31	-4.31	8.53	1.00	630	1219
Grassland : Peripheral	0.94	2.84	-4.53	6.78	1.00	435	1045
Road : Peripheral	-0.46	3.17	-6.56	5.92	1.00	607	1581
Rock : Peripheral	2.77	3.43	-4.01	9.58	1.00	683	1863
Woodland : Peripheral	0.72	2.92	-4.88	6.63	1.00	528	1143
Camp : Number of neighbours	1.37	1.06	-0.69	3.54	1.00	797	1924
Farm : Number of neighbours	1.85	1.43	-1	4.65	1.00	1112	2490
Forest : Number of neighbours	1.98	1.04	-0.02	4.05	1.00	718	1612
Grassland : Number of neighbours	1.11	0.92	-0.7	2.89	1.00	606	1479
Road : Number of neighbours	1.03	1.01	-0.9	3.03	1.00	674	1469
Rock : Number of neighbours	0.77	1.01	-1.16	2.78	1.00	695	1365
Woodland : Number of neighbours	1.32	0.94	-0.51	3.16	1.00	635	1463
Camp : Time not engaged	0.15	0.18	-0.22	0.52	1.00	772	1445
Farm : Time not engaged	-0.29	0.22	-0.72	0.14	1.00	946	1867
Forest : Time not engaged	0.35	0.21	-0.07	0.77	1.00	869	1743

Grassland : Time not engaged	-0.02	0.16	-0.33	0.28	1.01	619	1169
Road : Time not engaged	0.02	0.18	-0.33	0.37	1.01	773	1538
Rock : Time not engaged	0.28	0.21	-0.12	0.7	1.00	920	1809
Woodland : Time not engaged	0.27	0.16	-0.05	0.58	1.01	639	1070
Date							
sd(Intercept)	1.99	0.38	1.22	2.74	1.00	1503	1983
ID							
sd(Intercept)	1.19	0.48	0.19	2.07	1.00	774	804
Family							
sigma	10.35	0.26	9.86	10.86	1.00	2305	2630

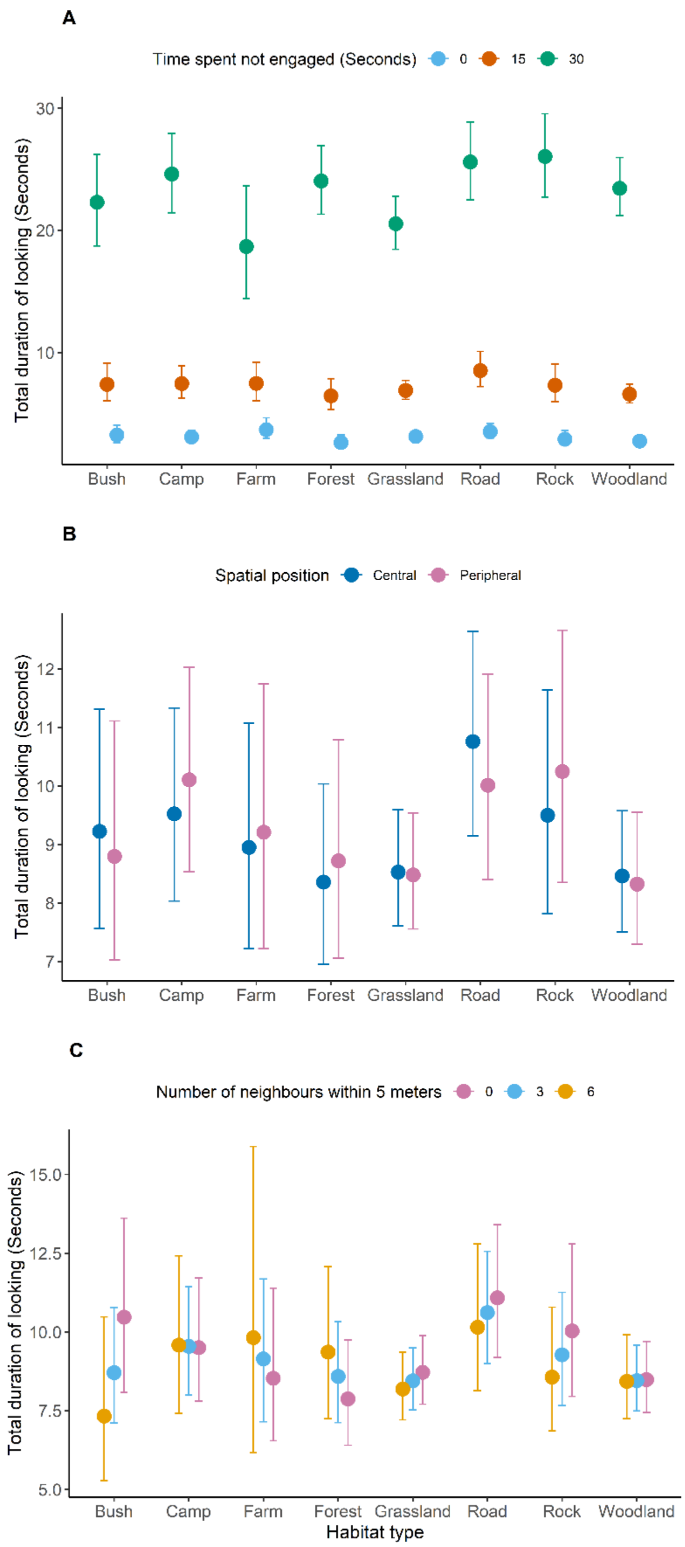

Figure S2. Conditional effects plots showing how the duration of looking bouts varied according to the interaction between habitat type and (panel A) time spent not engaged, (B) spatial position, and (C) number of neighbours within 5 meters. For time spent not engaged the conditional means and credible intervals (92.5% and 97.5% quantiles) are shown for specific values of time spent engaged (i.e., 0, 15, 30 seconds) for graphical purposes only. This was also done for number of neighbours at different values (0, 3, and 6 neighbours).

Table S21. Summary results for model exploring the relationship between the frequency of looking bouts and the interaction between home range familiarity at the location of the focal observation (inverted utilisation distribution) and several other reported risk factors, including time spent engaged, number of neighbours, spatial position. Also included are dominance rank of the focal animal, and visibility as these factors could influence the aforementioned relationship. The remainder of the population-level and group-level factors are the same as the minimal model.

	Estimate	Est.Error	l-95% CI	u-95% CI	Rhat	Bulk_ESS	Tail_ESS
Intercept	-2.25	0.1	-2.44	-2.05	1.00	1493	2456
Inverted utilisation distribution	0.04	0.09	-0.14	0.21	1.00	2553	2867
Spatial position (Peripheral)	0.06	0.03	0	0.12	1.00	3111	3212
Number of neighbours (5m)	-0.03	0.01	-0.05	-0.02	1.00	3371	3332
Time spent engaged	-0.01	0	-0.01	0	1.00	5340	3226
Rank	0	0	0	0.01	1.00	2036	3011
Visibility	0	0	0	0	1.00	4028	2836
Adolescent males	-0.3	0.11	-0.53	-0.08	1.00	1533	2641
Adult females	-0.16	0.06	-0.27	-0.05	1.00	1146	2045
Adult females (Infant contact)	-0.15	0.06	-0.28	-0.03	1.00	1370	2423
Adult females (Infant OS)	-0.05	0.12	-0.29	0.19	1.00	3402	2668
Adult males	-0.26	0.09	-0.44	-0.07	1.00	1181	1920
Juvenile-1 females	0.09	0.07	-0.06	0.23	1.00	1514	2311
Juvenile-1 males	0.02	0.08	-0.13	0.18	1.00	1298	2114
Juvenile-2 females	-0.04	0.06	-0.16	0.08	1.00	2107	2538
Juvenile-2 males	-0.05	0.07	-0.19	0.09	1.00	1092	1545
Juvenile-3 males	-0.11	0.09	-0.29	0.07	1.00	1252	2072
IUD : Peripheral	0.05	0.09	-0.11	0.23	1.00	3058	3336
IUD : Number of neighbours	0.03	0.02	-0.02	0.08	1.00	3236	3002
IUD : Time spent engaged	0.01	0	0	0.01	1.00	7150	2839
Date							
sd(Intercept)	0.17	0.02	0.14	0.21	1.00	1383	2349
ID							
sd(Intercept)	0.13	0.02	0.09	0.16	1.00	1617	2544

Table S22. Summary results for model exploring the relationship between the total duration of looking bouts and the interaction between home range familiarity at the location of the focal observation (inverted utilisation distribution) and several other reported risk factors, including time spent not engaged, number of neighbours, spatial position. Also included are dominance rank of the focal animal, and visibility as these factors could influence the aforementioned relationship. The remainder of the population-level and group-level factors are the same as the minimal model.

	Estimate	Est.Error	l-95% CI	u-95% CI	Rhat	Bulk_ESS	Tail_ESS
Intercept	-65.87	2.89	-71.67	-60.4	1.00	2413	2682
Inverted utilisation distribution	17.47	4.3	9.29	26.14	1.00	3689	2982
Spatial position (Peripheral)	-0.79	0.78	-2.32	0.71	1.00	4633	3480
Number of neighbours (5m)	-0.37	0.21	-0.78	0.03	1.00	4420	2785
Time spent not engaged	1.62	0.07	1.5	1.76	1.00	3244	3136
Rank	-0.01	0.02	-0.05	0.03	1.00	2887	2614
Visibility	0.14	0.02	0.11	0.18	1.00	7601	2975
Adolescent males	1.89	2	-2.06	5.8	1.00	1903	2540
Adult females	0.36	1.21	-2.02	2.75	1.00	1472	2204
Adult females (Infant contact)	1.46	1.43	-1.46	4.3	1.00	1713	2239
Adult females (Infant OS)	2.2	3.35	-4.41	8.5	1.00	4386	2856
Adult males	2.37	1.65	-0.89	5.63	1.00	1565	2162
Juvenile-1 females	-2.18	1.59	-5.28	0.93	1.00	2104	2653
Juvenile-1 males	-1.86	1.52	-4.86	1.15	1.00	1880	2538
Juvenile-2 females	-0.38	1.48	-3.36	2.47	1.00	2199	2749
Juvenile-2 males	2.38	1.33	-0.25	4.98	1.00	1579	2639
Juvenile-3 males	1.27	1.73	-2.1	4.69	1.00	1662	2668
IUD : Peripheral	-2.55	2.14	-6.61	1.62	1.00	4441	3167
IUD : Number of neighbours	-1.01	0.55	-2.06	0.07	1.00	4104	3100
IUD : Time spent not engaged	-0.49	0.15	-0.79	-0.21	1.00	4126	3230
Date							
sd(Intercept)	2.01	0.38	1.28	2.75	1.00	1617	2030
ID							
sd(Intercept)	1.2	0.47	0.18	2.05	1.00	921	1047
Family							
sigma	10.4	0.25	9.94	10.91	1.00	2549	3228

Table S23. Summary results for model exploring the relationship between the frequency of looking bouts and the interaction between home range familiarity at the location of the focal observation (core, frequently used, and boundary areas) and several other reported risk factors, including time spent engaged, number of neighbours, spatial position. Also included are dominance rank of the focal animal, and visibility as these factors could influence the aforementioned relationship. The remainder of the population-level and group-level factors are the same as the minimal model.

	Estimate	Est.Error	l-95% CI	u-95% CI	Rhat	Bulk_ESS	Tail_ESS
Intercept	-2.26	0.1	-2.47	-2.06	1.00	1455	2136
Home range familiarity (Core)	-0.07	0.05	-0.17	0.04	1.00	2231	2654
Home range familiarity (Frequent)	0.06	0.05	-0.04	0.16	1.00	2311	3091
Spatial position (Peripheral)	0.07	0.04	0	0.15	1.00	2176	2401
Number of neighbours (5m)	-0.01	0.01	-0.04	0.01	1.00	2297	3095
Time spent engaged	-0.01	0	-0.01	0	1.00	6223	3713
Rank	0	0	0	0.01	1.00	2186	2674
Visibility	0	0	0	0	1.00	4019	3013
Adolescent males	-0.31	0.11	-0.53	-0.09	1.00	1608	2879
Adult females	-0.16	0.06	-0.28	-0.05	1.00	990	2045
Adult females (Infant contact)	-0.15	0.07	-0.28	-0.02	1.00	1342	2583
Adult females (Infant OS)	-0.04	0.12	-0.29	0.19	1.00	3034	2815
Adult males	-0.27	0.09	-0.44	-0.08	1.00	1162	2043
Juvenile-1 females	0.08	0.07	-0.07	0.22	1.00	1586	1927
Juvenile-1 males	0.02	0.08	-0.13	0.17	1.00	1153	1962
Juvenile-2 females	-0.04	0.06	-0.16	0.07	1.00	1875	2461
Juvenile-2 males	-0.06	0.07	-0.19	0.08	1.00	1100	1862
Juvenile-3 males	-0.12	0.09	-0.29	0.06	1.00	1442	2332
Core : Peripheral	-0.02	0.05	-0.12	0.08	1.00	2300	2735
Frequent : Peripheral	-0.08	0.05	-0.18	0.02	1.00	2349	2380
Core : Number of neighbours	-0.03	0.02	-0.06	0	1.00	2502	2637
Frequent : Number of neighbours	-0.04	0.02	-0.07	-0.01	1.00	2559	2953
Core : Time spent engaged	-0.01	0	-0.01	0	1.00	5913	3441
Frequent : Time spent engaged	0	0	-0.01	0	1.00	6103	3141
Date							
sd(Intercept)	0.17	0.02	0.14	0.21	1.00	1478	2068
ID							
sd(Intercept)	0.12	0.02	0.09	0.16	1.00	1755	2829

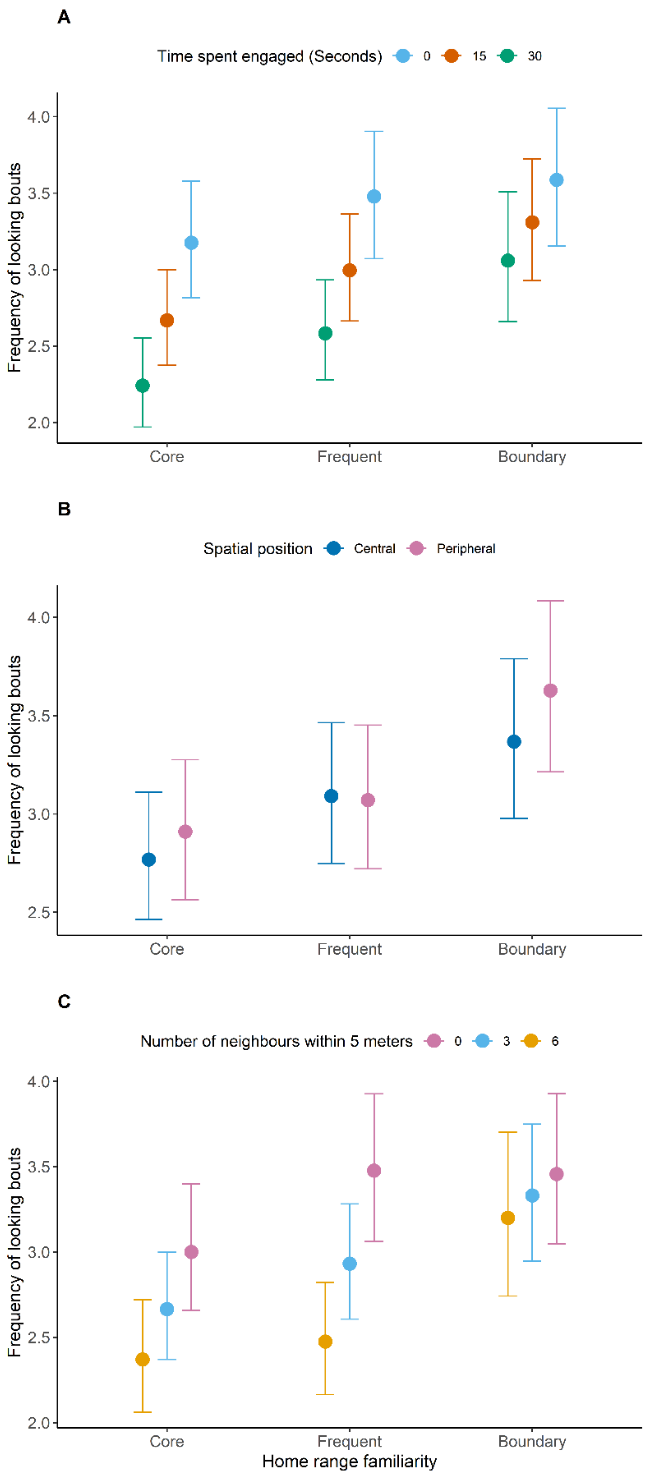

Figure S3. Conditional effects plots showing how the frequency of looking bouts varied according to the interaction between home range familiarity (core, frequently used, and boundary areas) and time spent engaged (panel A), spatial position (B), and number of neighbours within 5 meters (C). For time spent engaged the conditional means and credible intervals (2.5% and 97.5% quantiles) are shown for specific values of time spent engaged (i.e., 0, 15, 30 seconds) for graphical purposes only. This was also done for number of neighbours at different values (0, 3, and 6 neighbours).

Table S24. Summary results for model exploring the relationship between the total duration of looking bouts and the interaction between home range familiarity at the location of the focal observation (core, frequently used, and boundary areas) and several other reported risk factors, including time spent not engaged, number of neighbours, spatial position. Also included are dominance rank of the focal animal, and visibility as these factors could influence the aforementioned relationship. The remainder of the population-level and group-level factors are the same as the minimal model.

	Estimate	Est.Error	l-95% CI	u-95% CI	Rhat	Bulk_ESS	Tail_ESS
Intercept	-62.58	3.05	-68.77	-56.95	1.00	1213	2304
Home range familiarity (Core)	-14.58	2.51	-19.57	-9.73	1.00	1575	2631
Home range familiarity (Frequent)	-8.17	2.36	-12.88	-3.52	1.00	1530	2246
Spatial position (Peripheral)	-0.62	0.94	-2.41	1.27	1.00	1904	2622
Number of neighbours (5m)	-0.35	0.28	-0.9	0.19	1.00	1583	2141
Time spent not engaged	1.51	0.07	1.38	1.66	1.00	1496	2195
Rank	-0.01	0.02	-0.05	0.03	1.00	2048	2633
Visibility	0.14	0.02	0.11	0.17	1.00	4575	3411
Adolescent males	1.87	2.03	-2.2	5.91	1.00	1387	2303
Adult females	0.37	1.21	-1.96	2.73	1.00	859	1488
Adult females (Infant contact)	1.52	1.44	-1.4	4.32	1.00	1032	1585
Adult females (Infant OS)	2.64	3.25	-3.74	8.89	1.00	2598	2829
Adult males	2.36	1.63	-0.85	5.57	1.00	1002	1733
Juvenile-1 females	-2.34	1.58	-5.52	0.76	1.00	1144	1939
Juvenile-1 males	-1.95	1.55	-4.9	1.13	1.00	1045	1917
Juvenile-2 females	-0.33	1.45	-3.15	2.55	1.00	1213	1745
Juvenile-2 males	2.43	1.37	-0.28	5.16	1.00	871	1621
Juvenile-3 males	1.31	1.69	-2	4.6	1.00	1092	1850
Core : Peripheral	1.37	1.28	-1.09	3.82	1.00	2174	2825
Frequent : Peripheral	-0.06	1.31	-2.6	2.51	1.00	2186	2994
Core : Number of neighbours	0.53	0.36	-0.19	1.23	1.00	1827	2228
Frequent : Number of neighbours	0.19	0.36	-0.49	0.91	1.00	1865	2478
Core : Time spent not engaged	0.45	0.09	0.28	0.62	1.00	1798	2319
Frequent : Time spent not engaged	0.25	0.08	0.09	0.42	1.00	1651	2469
Date							
sd(Intercept)	2	0.38	1.28	2.76	1.00	1400	1922
ID							
sd(Intercept)	1.09	0.5	0.1	2.03	1.01	668	702
Family							
sigma	10.43	0.25	9.94	10.94	1.00	1908	2373

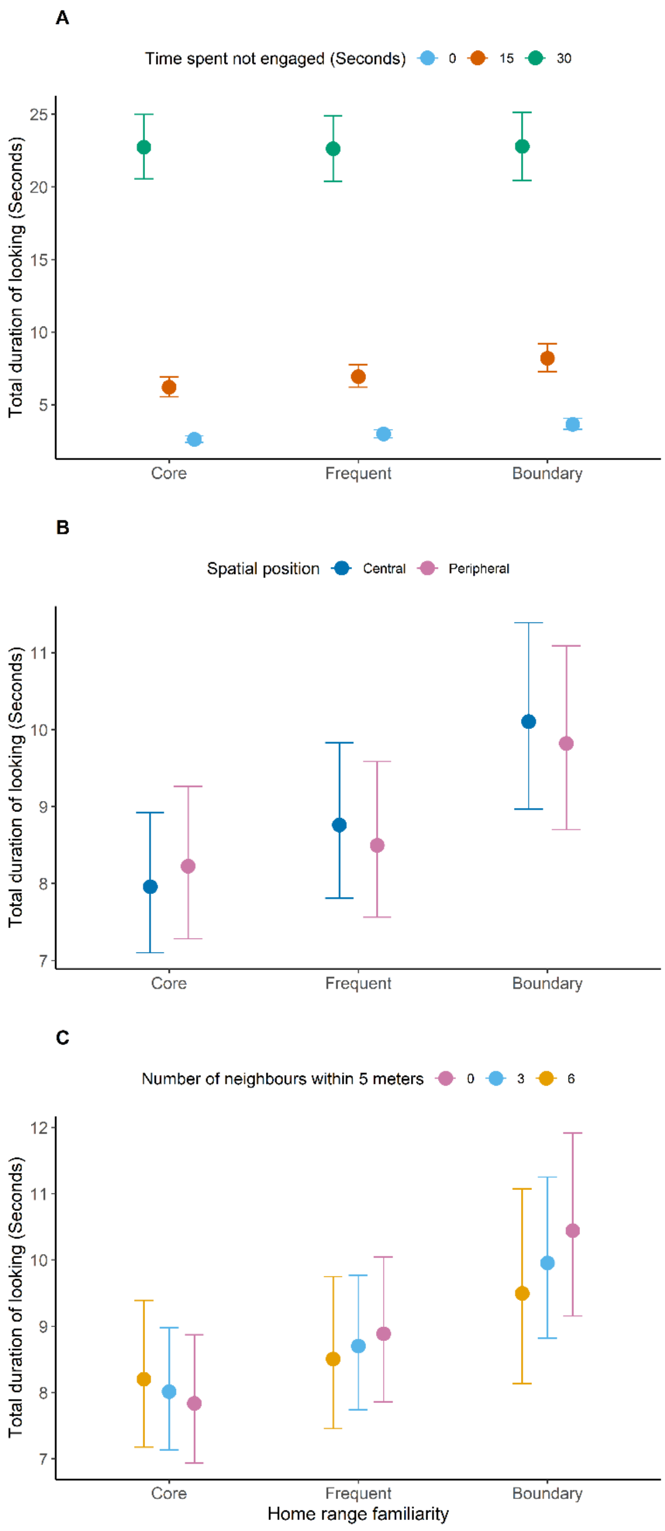

Figure S4. Conditional effects plots showing how the duration of looking bouts varied according to the interaction between home range familiarity (core, frequently used, and boundary areas) and time spent engaged (panel A), spatial position (B), and number of neighbours within 5 meters (C). For time spent engaged the conditional means and credible intervals (2.5% and 97.5% quantiles) are shown for specific values of time spent engaged (i.e., 0, 15, 30 seconds) for graphical purposes only. This was also done for number of neighbours at different values (0, 3, and 6 neighbours).

Table S25. Summary results for model exploring the relationship between the frequency of looking bouts and the interaction between probability of encountering another group (at the focal observation location) and several other reported risk factors, including time spent engaged, number of neighbours, spatial position. Also included are dominance rank of the focal animal, and visibility as these factors could influence the aforementioned relationship. The remainder of the population-level and group-level factors are the same as the minimal model.

	Estimate	Est.Error	l-95% CI	u-95% CI	Rhat	Bulk_ESS	Tail_ESS
Intercept	-2.21	0.1	-2.4	-2.02	1.00	1460	2367
Risk of encountering another group (WE)	-0.57	0.27	-1.1	-0.06	1.00	2425	2791
Spatial position (Peripheral)	0.05	0.03	0	0.11	1.00	3155	2837
Number of neighbours (5m)	-0.06	0.01	-0.08	-0.05	1.00	3286	3159
Time spent engaged	-0.01	0	-0.01	-0.01	1.00	4222	3579
Rank	0	0	0	0.01	1.00	1794	2748
Visibility	0	0	0	0	1.00	4516	3055
Adolescent males	-0.3	0.11	-0.52	-0.09	1.00	1456	2317
Adult females	-0.16	0.06	-0.28	-0.04	1.01	987	1723
Adult females (Infant contact)	-0.16	0.07	-0.28	-0.03	1.00	1229	1824
Adult females (Infant OS)	-0.05	0.12	-0.29	0.18	1.00	2684	2901
Adult males	-0.26	0.09	-0.44	-0.08	1.00	1086	1842
Juvenile-1 females	0.08	0.07	-0.07	0.22	1.00	1397	2145
Juvenile-1 males	0.02	0.08	-0.14	0.17	1.00	1196	1904
Juvenile-2 females	-0.04	0.06	-0.17	0.08	1.00	1605	2308
Juvenile-2 males	-0.05	0.07	-0.19	0.08	1.00	1029	1781
Juvenile-3 males	-0.11	0.09	-0.29	0.07	1.00	1249	1976
WE : Peripheral	-0.16	0.27	-0.68	0.36	1.00	2632	3094
WE : Number of neighbours	0.32	0.08	0.16	0.47	1.00	2765	2771
WE : Time spent engaged	0.03	0.01	0.01	0.06	1.00	4721	3189
Date							
sd(Intercept)	0.16	0.02	0.13	0.2	1.00	1432	2373
ID							
sd(Intercept)	0.12	0.02	0.09	0.16	1.00	1797	2689

Table S26. Summary results for model exploring the relationship between the total duration of looking bouts and the interaction between probability of encountering another group (at the focal observation location) and several other reported risk factors, including time spent not engaged, number of neighbours, spatial position. Also included are dominance rank of the focal animal, and visibility as these factors could influence the aforementioned relationship. The remainder of the population-level and group-level factors are the same as the minimal model.

	Estimate	Est.Error	l-95% CI	u-95% CI	Rhat	Bulk_ESS	Tail_ESS
Intercept	-71.46	2.91	-77.39	-65.95	1.00	3035	2824
Risk of encountering another group (WE)	23.75	12.12	-0.11	46.77	1.00	4147	2737
Spatial position (Peripheral)	-0.68	0.69	-2.08	0.63	1.00	4836	3615
Number of neighbours (5m)	0	0.18	-0.36	0.35	1.00	5068	3072
Time spent not engaged	1.77	0.07	1.65	1.91	1.00	3594	2756
Rank	-0.01	0.02	-0.05	0.03	1.00	3599	3080
Visibility	0.14	0.02	0.11	0.17	1.00	6541	3063
Adolescent males	2.32	2.07	-1.6	6.38	1.00	2377	2921
Adult females	0.46	1.22	-1.94	2.88	1.00	1855	2434
Adult females (Infant contact)	1.65	1.44	-1.22	4.42	1.00	2090	2720
Adult females (Infant OS)	2.31	3.16	-3.91	8.34	1.00	4777	2831
Adult males	2.31	1.68	-0.98	5.64	1.00	2165	2578
Juvenile-1 females	-2.09	1.62	-5.27	1.13	1.00	2571	3123
Juvenile-1 males	-1.82	1.56	-4.76	1.39	1.00	2380	3006
Juvenile-2 females	-0.28	1.44	-3.19	2.46	1.00	2658	3302
Juvenile-2 males	2.45	1.37	-0.25	5.1	1.00	1995	2515
Juvenile-3 males	1.36	1.74	-2.07	4.77	1.00	2136	2820
WE : Peripheral	8.79	7.03	-5.16	22.31	1.00	4893	3038
WE : Number of neighbours	-1.37	1.93	-5.21	2.44	1.00	4745	2828
WE : Time spent engaged	-0.44	0.41	-1.23	0.38	1.00	4544	2728
Date							
sd(Intercept)	1.99	0.37	1.27	2.74	1.00	1630	2596
ID							
sd(Intercept)	1.25	0.49	0.17	2.16	1.00	963	923
Family							
sigma	10.37	0.24	9.9	10.86	1.00	3943	3218

Table S27. Summary results for model exploring the relationship between the frequency of looking bouts and the interaction between the individual tolerance level (of the focal animal) and several other reported risk factors, including time spent engaged, observer distance, and observer movement. The remainder of the population-level and group-level factors are the same as the minimal model.

	Estimate	Est.Error	l-95% CI	u-95% CI	Rhat	Bulk_ESS	Tail_ESS
Intercept	-2.15	0.09	-2.32	-1.98	1.00	2591	2942
Tolerance	0.18	0.08	0.02	0.35	1.00	3677	2981
Observer distance (meters)	0.01	0.01	-0.01	0.02	1.00	4674	3722
Time spent engaged	-0.01	0	-0.02	-0.01	1.00	5930	3355
Observer movement (Yes)	0.17	0.06	0.04	0.29	1.00	5265	3091
Adolescent males	-0.44	0.1	-0.64	-0.24	1.00	2948	3401
Adult females	-0.19	0.06	-0.31	-0.08	1.00	2056	2365
Adult females (Infant contact)	-0.21	0.07	-0.34	-0.08	1.00	2245	3186
Adult females (Infant OS)	-0.08	0.12	-0.32	0.15	1.00	3588	3075
Adult males	-0.4	0.08	-0.55	-0.25	1.00	2282	2815
Juvenile-1 females	0.07	0.08	-0.07	0.22	1.00	2569	2923
Juvenile-1 males	-0.1	0.08	-0.25	0.06	1.00	1952	2364
Juvenile-2 females	-0.06	0.06	-0.19	0.06	1.00	2941	2856
Juvenile-2 males	-0.14	0.07	-0.28	0	1.00	1722	2540
Juvenile-3 males	-0.23	0.08	-0.39	-0.06	1.00	2473	2994
Tolerance : Observer distance	-0.04	0.01	-0.06	-0.02	1.00	4176	3424
Tolerance : Time spent engaged	0	0	0	0.01	1.00	6394	3403
Tolerance : Observer movement (Yes)	-0.07	0.07	-0.21	0.08	1.00	5410	2970
Date							
sd(Intercept)	0.16	0.02	0.13	0.2	1.00	1157	2459
ID							
sd(Intercept)	0.13	0.02	0.1	0.17	1.00	1556	2318

Table S28. Summary results for model exploring the relationship between the total duration of looking bouts and the interaction between the individual tolerance level (of the focal animal) and several other reported risk factors, including time spent not engaged, observer distance, and observer movement. The remainder of the population-level and group-level factors are the same as the minimal model.

	Estimate	Est.Error	l-95% CI	u-95% CI	Rhat	Bulk_ESS	Tail_ESS
Intercept	-66.09	3.08	-72.32	-60.2	1.00	1962	2403
Tolerance	5.52	2.66	0.37	10.66	1.00	2666	2903
Observer distance (meters)	0.2	0.19	-0.18	0.58	1.00	3394	3333
Observer movement (Yes)	2.38	1.82	-1.27	5.92	1.00	4250	3156
Time spent not engaged	1.93	0.09	1.76	2.11	1.00	2213	2501
Adolescent males	3.17	1.82	-0.45	6.72	1.00	2368	3273
Adult females	0.46	1.27	-2.06	2.94	1.00	1395	2405
Adult females (Infant contact)	1.77	1.51	-1.22	4.76	1.00	1888	2930
Adult females (Infant OS)	2.52	3.33	-3.99	9	1.00	4461	3200
Adult males	3.66	1.39	0.9	6.33	1.00	1642	2128
Juvenile-1 females	-2.04	1.61	-5.19	1.06	1.00	1894	2490
Juvenile-1 males	-1.66	1.57	-4.74	1.39	1.00	1617	2678
Juvenile-2 females	0.04	1.5	-2.94	2.89	1.00	1904	2381
Juvenile-2 males	3.36	1.39	0.67	6.07	1.00	1427	2533
Juvenile-3 males	2.48	1.6	-0.64	5.67	1.00	1697	2599
Tolerance : Observer distance	-0.17	0.24	-0.65	0.3	1.00	3332	3184
Tolerance : Observer movement (Yes)	-1.95	2.08	-5.91	2.22	1.00	4217	3107
Tolerance : Time spent not engaged	-0.2	0.08	-0.37	-0.04	1.00	3234	3145
Date							
sd(Intercept)	2.1	0.39	1.35	2.89	1.00	1677	1986
ID							
sd(Intercept)	1.23	0.49	0.19	2.15	1.01	834	804
Family							
sigma	10.58	0.26	10.1	11.1	1.00	2882	2802

Text S2. Age-sex class categories and descriptions

Female baboons:

Adult female – Attainment of full body size, either cycling regularly, pregnant or lactating. Nipples also enlarge and elongated from suckling infants.

Adult female with infant contact – Adult females with dependent infants (black pelage and natal/pink skin colouration) within immediate sight. In open areas with high visibility, this could extend upto 10 meters as long as the infant was not isolated/exposed, e.g., had other baboons within 5 meters or was playing with other infants or juveniles. In areas of dense vegetation, the adult female needed to have direct line of sight to her infant to be considered 'in contact', as such, infants were occasionally considered not in contact with their mothers despite being relatively close.

Adult female with infant OS (out of sight) – Adult females with dependent infants beyond their immediate sight. This could be infants obscured by dense vegetation, beyond 10 meters away, or between 5 and 10 meters but with no other individuals within 5 meters, i.e., exposed/isolated.

ADF (Adolescent Female) – Nearly adult female size, with the onset of the first sexual swellings. If visible, nipples are much smaller and button-like than that of an adult female.

Male baboons:

AM (Adult male) – All secondary sexual characteristics fully grown, musculature (most noticeably in chest and rump) expands to full adult size.

ADM (Adolescent Male) – Massive growth in secondary sexual characteristics; testes expand, canines and mane grow longer, body size increases to near that of an adult male.

J3M (Juvenile 3 Males Only) – Body size at least that of an adult female, muzzle further extended to nearly that of an adult male. Testes start to expand and are clearly visible. Mane becomes noticeable.

Juvenile baboons of both sexes:

J2M/F (Juvenile 2) – Little demarcation from previous period, with greater body size. Hair becomes darker, changing to a more adult grey/brown colouration.

J1M/F (Juvenile 1) – Little demarcation from infants, but fully weaned and nutritionally independent. Muzzle starts becoming more elongated and pronounced. Pelage is still lighter than in juvenile 2. Male/female distinction based on genitalia and noticeable absence/presence of a separation in the callosities.

Text S3: Pilot study to validate methodology

Methods for identifying the ideal focal observation duration

We undertook a pilot-field study to identify the appropriate focal observation length for this study and assess the feasibility of some of the contextual variables. The pilot-field study used the methodology detailed in the Main Text (subject to minor changes detailed below). For pilot work, a maximum focal observation duration was set at 4 minutes, as this represented the higher end of what we felt was likely to be achievable (based on experience and findings from [37]).

Results

Successful focal observation duration

Only 22 of 155 focal observations reached the 4-minute maximum observation length, with 12 of these observations in time-period 4 (15:00 – 17:40). Aborted focal observations were usually due to animals moving out of sight (e.g., cliffs or dense vegetation). Sixty-two of the 155 total observations achieved a minimum duration of 90 seconds (40% success rate), whilst 60-second focals had a 65% success rate (100 observations) - with at least one observation on each randomly chosen individual (25 individuals) within each time-period (i.e., T1, T2, T3 and T4). The number of samples reaching 60 seconds increased in the latter two time-periods (fig S1), whilst focal observation duration generally increased towards the end of the day, likely due to baboons performing more stationary behaviours such as grooming and resting at these times.

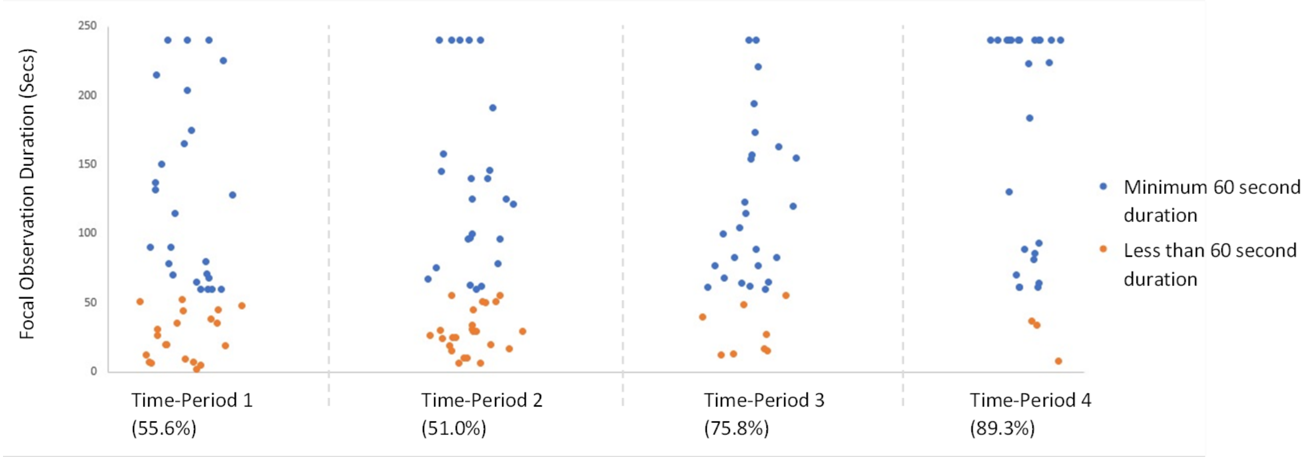

Fig S5. Focal observation duration (seconds) in each time-period. Each dot represents a single focal observation. Percentages in parentheses represent the relative success rate for 60-second focals in each time-period.

These findings suggested that selecting a focal observation duration of 4-minutes would likely lead to a high-degree of aborted samples, but that even 60 seconds may bias observations towards stationary behaviours or 'relaxed' scenarios. We therefore, explored the success of 30-second observations. We found a 79.4% success rate (123 observations), with an even spread of observations across time periods (T1 and T2: 34, T3: 28, T4: 27), suggesting that 30-second observations represented the best duration for maximising the number of successful samples, recording a diverse range of behaviours and scenarios, and minimising observer fatigue. We therefore explored whether the temporal organisation of looking bouts was affected by the choice of 60-second and 30-second observation lengths.

The distribution of looking bout durations gathered from 60s focal observations

Looking bout durations were extracted from the 60-second focal observations, yielding 296 separate looking bouts. The distribution of bout durations had a strong positive-skew (fig S2). Of these bouts only 6 spanned the entire 60-second focal observation, indicating that truncating looking bouts happened rarely.

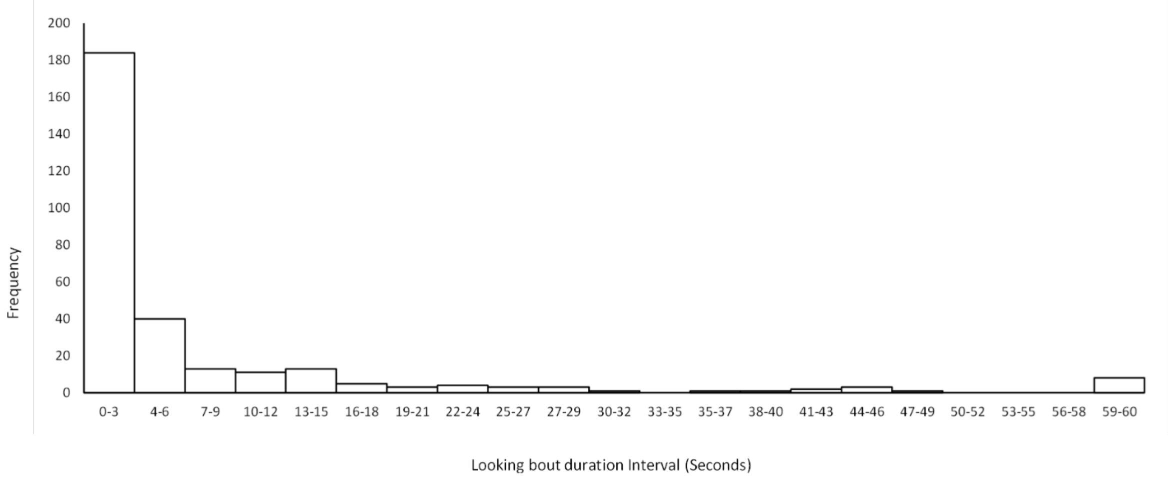

Fig S6. Frequency histogram showing the distribution of looking bout durations in 3-second intervals across 60-second focal observations.

The distribution of bout durations between 1 and 3 seconds (fig S3) had a clear positive-skew, showing little variation in the utilisation. Conversely, the distribution of looking between 0 and 1 seconds reflected a unimodal and relatively symmetric distribution. This highlighted that this baboon group consistently used short rapid looking bouts lasting < 1 second.

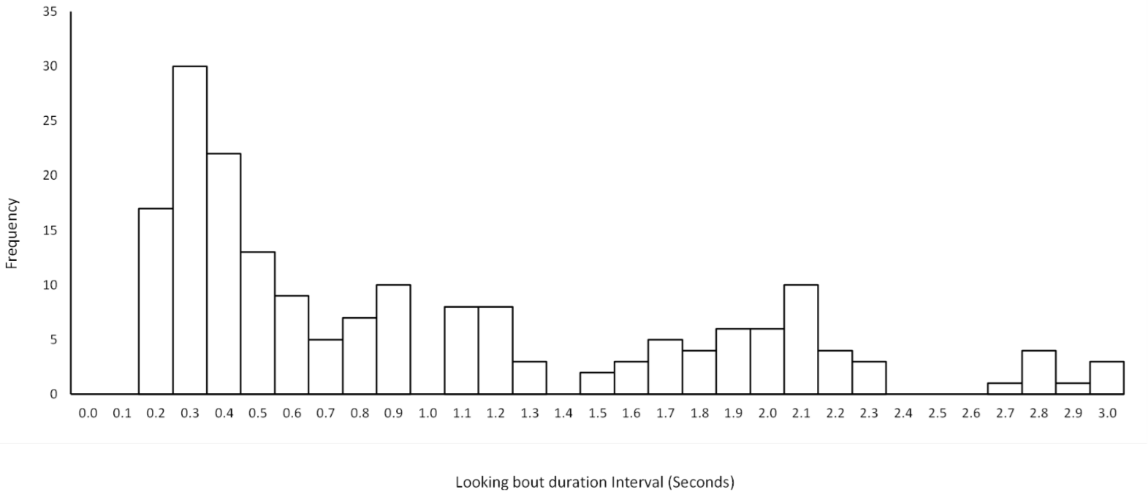

Fig S7. Frequency histogram showing the distribution of looking bout durations of less than 3-seconds, each bar represents a tenth of a second.

The distribution of looking bout durations gathered from 30s focal observations

One-hundred and sixty-six individual looking bouts were recorded across 123 30-second focal observations, with the distribution having a strong positive-skew (Fig S4). Of these bouts, 16 bouts (9.3% of the total number of individual bouts) spanned the entire 30-second focal observation, creating a small increase towards the tail of the distribution and truncating some bouts to greater extent than the 60-second methodology (2.3% of bouts truncated for 60-second observations).

However, the increase in individual bouts reaching observation length in 30-second focals was still minimal, and did not alter the skew or distribution of individual looking bouts. It seems likely that this is the minimum threshold though, as reducing the focal observation by another 10-seconds would have shifted the data towards a bi-modal distribution.

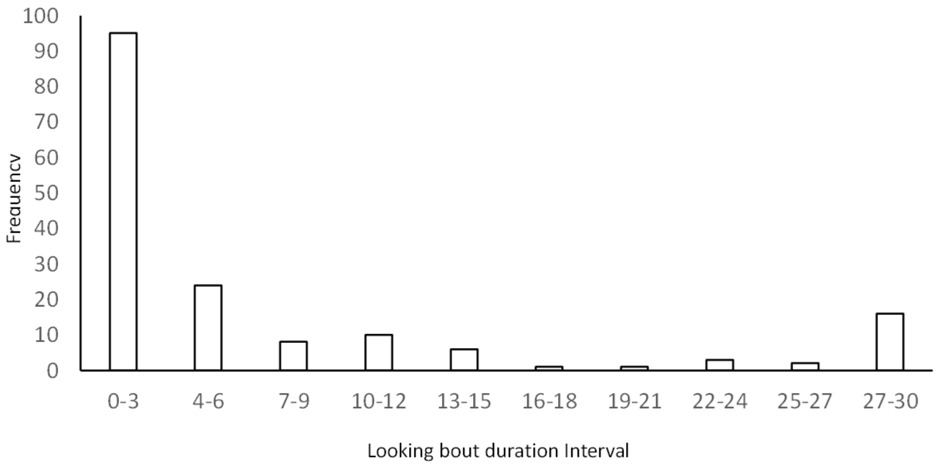

Fig S8. Frequency histogram showing the distribution of vigilance bout durations in 3-second intervals across 30-second focal observations.

The distribution of looking bout durations between 1 and 3 seconds (fig S5) had a clear positive-skew, showing little variation. The distribution of looking between 0 and 1 second reflected a unimodal and relatively symmetric distribution. Both distributions varied minimally to the distributions found in 60-second observations. Therefore, we selected 30-second observations for our study as our pilot work revealed that this length allowed us to gather robust data on the temporal organisation of looking whilst minimising the chance of regularly truncating looking bouts.

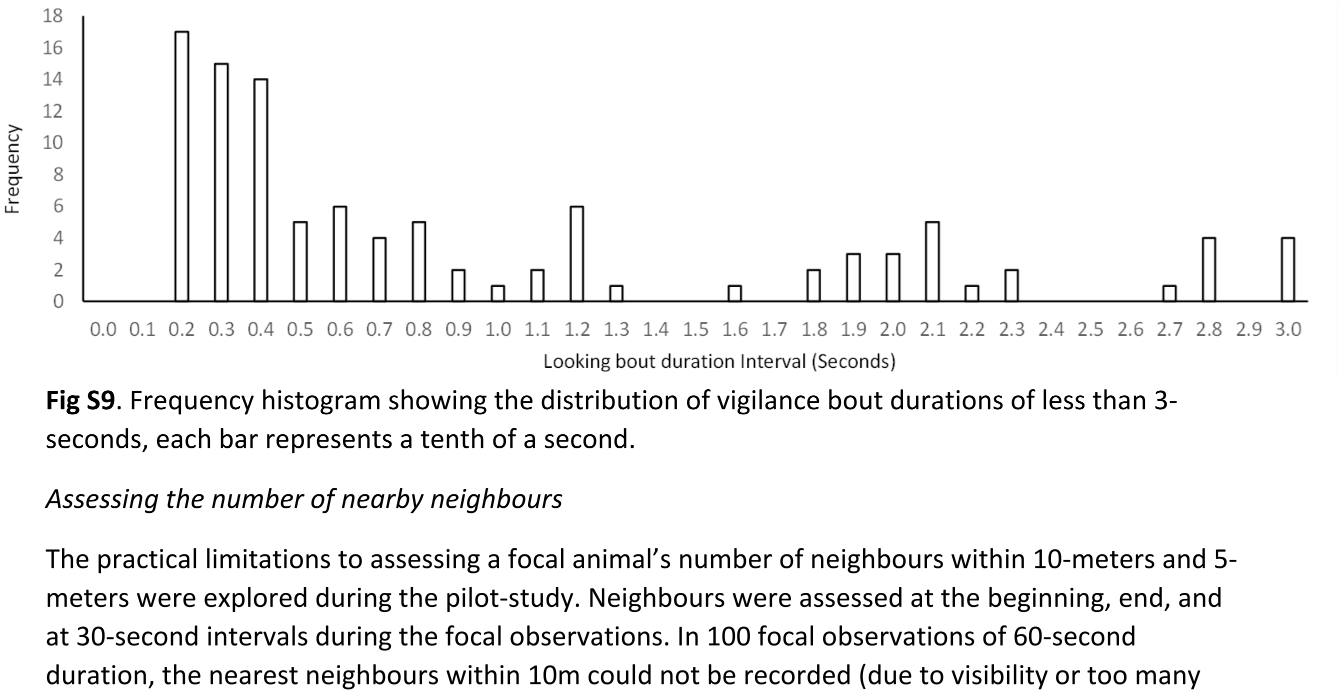

Fig S9. Frequency histogram showing the distribution of vigilance bout durations of less than 3-seconds, each bar represents a tenth of a second.

Assessing the number of nearby neighbours

The practical limitations to assessing a focal animal's number of neighbours within 10-meters and 5-meters were explored during the pilot-study. Neighbours were assessed at the beginning, end, and at 30-second intervals during the focal observations. In 100 focal observations of 60-second duration, the nearest neighbours within 10m could not be recorded (due to visibility or too many individuals to count reliably using an instantaneous point sampling method) 7 times out of a total of 300 nearest neighbour scans. There were no unsuccessful 5 meter nearest neighbour scans. We therefore chose 5 meters as it reduced observer fatigue and eliminated the chances for errors.

Text S4: Methods for assessing focal habitat visibility

To assess whether the observer (AA) could assess habitat visibility as a percentage for each focal observation, AA generated 20 random GPS locations within the baboon's core ranging area. At each GPS location horizontal habitat visibility was assessed in each cardinal and intercardinal (i.e., north-east, south-east, etc.,) direction to both 5 meter and 10-meter distances, each assessment was completed at both 50cm (to reflect baboon eye-level on the ground) and 1.5 meters (to reflect animal's foraging in trees). At each direction, distance, and height a photograph was taken (from the GPS location) towards a 1x1 meter checkerboard with 225 squares, generating 32 checkerboard photos for each GPS location. The number of squares visible in each photo was then counted post-hoc. For each plot, the percentage of visible squares was calculated as a percentage of total squares for cardinal and intercardinal directions separately, and for each height separately, producing a total of four visibility assessments for each plot (i.e., 50cm cardinal direction, 1.5m cardinal, 50cm intercardinal, 1.5m intercardinal), 80 assessments across all 20 plots.

At each plot AA visually assessed visibility to 5 and 10 meters at 50cm and 1.5m from the ground, taking into account numerous visual obstructions that could hinder a baboon's view to those distances in a 360-degree view. To assess the validity of AA's assessments, the cardinal and intercardinal assessments were plotted along with a regression line calculated from a linear model exploring their relationship (fig S6). The predictions intervals for the cardinal vs intercardinal relationship were then calculated at the 95% level and added to the plot to represent upper and lower prediction intervals, AA's visual assessments were then added. Only two of AA's visual assessments were outside of the prediction interval calculated from checkboard assessments, suggesting that 97.5% of these assessments were within the prediction bounds. AA's visual assessments may have been very slightly higher than checkerboard assessments due to AA being able to incorporate more information than the photographic method. Given that we were also assessing n the number of neighbours within 5-meters, we therefore decided to assess the visibility to 5 meters for each of our focal observations in the main study too, thus aligning the assessments.

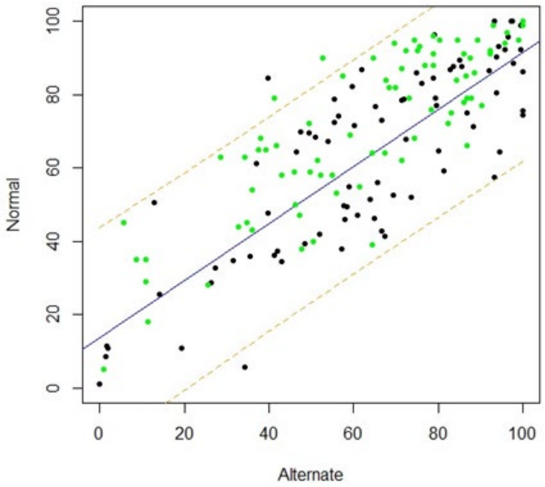

Figure S10. Regression between checkerboard visibility assessments of cardinal ('normal') direction and the alternate checkboard placements (intercardinal). Black dots represent the checkerboard visibility assessments, and the blue line is the linear model relationship between these assessments. The dashed orange lines represent the prediction intervals calculated at 95%. The green dots represent the visual assessments of the same locations by the observer, highlighting the observer's assessment fall within the prediction interval of the checkerboard assessments.

Text S5. Descriptions of the various habitat types categorised in this study

Habitat type – the Lajuma field site and surrounding properties contain a range of habitat types that can be broadly classified into several categories: forest, woodland, bush, camp, farm, grassland, and rocky areas. These habitat types vary in their respective structure and potential threats.

Forest: An area composed largely of trees with overlapping crowns forming 60-100% cover. Trees will be mostly tall providing extensive and near continuous shade.

Woodland: Canopy is more open than forest, with 25-60% cover, allowing sunlight to penetrate between the trees. Woodlands may support an understory of shrubs, herbs, or grasses.

Bush: An area where shrubs or are the dominant vegetation. A shrub is a woody perennial plant, smaller than a tree, with several major branches arising near the base of the stem. Areas of extensive tree regeneration, i.e., saplings, can also qualify as bush.

Grassland or savannah: Open area covered predominantly with grass. These areas may be devoid of trees entirely but can also contain widely spaced trees with a minimum of 5% cover to a maximum of 25% cover. Also included marshland which was exclusively found in flat regions along permanent water streams on peat. Vegetation components of marshlands include reeds, sedges, and grasses.

Rock: Areas where ground predominantly consists of rocks and boulders, rather than soil. Typically open and devoid of vegetation, as such, visibility can be higher in places, which can allow very distant threats to be detected.

Road: Dirt roads that run through the study area. Very little traffic (less than 5 vehicles a day) and always at low speeds (less than 10mph). Road use often offers a localised enhancement in visibility for the baboons.

Camp: Used or disused human settlements on Lajuma and neighbouring properties. Occasionally chased away from properties but usually very passive interactions with humans in these areas. Likely predation risk is lower, but conflict with domestic dogs is most common in these locations.

Farm: Ottosdal Macadamia farm or area around Ottosdal farmhouse, a consistent site for conflict with humans.

Text S6. Defining clique membership using the spinglass algorithm

The spinglass algorithm allows cliques to be formed even when certain members are not consistently observed interacting but are grouped as they share close associates. For example, A – B, C -D, E - A, F – C, may all be very consistent dyads, but observations of B – C, D – A, F – A, etc., are rare. The spinglass algorithm can detect the clusters, i.e., connection chains, placing these individuals in the same clique. This meant that instead having numerous mother-offspring only cliques, we had cliques involving several related adult females and their associated offspring, and the adult males that consistently associated with them.

We used dyadic grooming interactions as the association measure for community detection, such that individuals are unlikely to be considered a threat if they share consistent grooming interactions. We used ad libitum sampling to record grooming interactions, for all grooming events the identity and direction of the interaction was recorded and later used to create weighted, directed matrices for 2018 and 2019 separately. This grooming data was collected outside of focal observations allowing observer effects on inter-individual association patterns to be minimised as AA could stand further away when collecting the grooming information than for focal sampling (see [49]).

Clique membership was updated for each year to reflect changes in affiliative tendencies due to reproductive cycles, consortships, births, and deaths. We decided to keep grooming information recorded during consortships in these analyses as they reflected important aspects of the baboons ongoing behaviour and likely had a key role in social threat perception, e.g., adult females likely do not consider a regular consort partner a threat on a consistent basis. On two occasions adult males were grouped in the same clique. Their focal observations were updated such that they should still consider the other male to be a threatening individual.

Text S7: Detailed methods for constructing spatial risk layers for pre-emptive vigilance hypotheses

Between February 2015 and July 2019, AA and research assistants collected ranging data for the study group and encounter data for all interactions with other groups of baboons or lone males (i.e., foreign individuals). Ranging data consisted of marking GPS points every 20 minutes throughout full day follows, e.g., 06:00, 06:20. 06:40 etc, producing n=11936 GPS points. Encounters with other groups of baboons were marked using a GPS when the events occurred (n=240). Some of these events could be very brief, e.g., sighting a lone male on a cliff, or remaining in proximity to another group for several hours. During the longer episodes, additional GPS points were only updated if the groups became visually separated by some obstruction, e.g., mist, woodland, cliff, and then encountered each other again at least 5 minutes later. Additional GPS points were also recorded if the status of the encounter changed, e.g., a passive encounter became active.

All ranging GPS points were entered into local convex hulls analysis (T-LoCoH)[53] to calculate home range utilisation distribution (UD) across all years (i.e., one consolidated home range). T-LoCoH generalises the local convex hull procedure (i.e., LoCoH[54]) to incorporate time and space into local hull construction. We used the fixed-*k* method (set to 24) to identify the nearest neighbours of each point and set the time scaled-distance metric set to 0.01, to ensure correct construction of isopleths[54]. These values were selected with the aid of the graphical procedures available in the *tlocoh* package which allowed assessment of how different values minimised spurious holes and captured density gradients within the home range[53].

For the home-range familiarity continuous variable, the utilisation distribution was defined as the 99% isopleth and intensity of use calculated at 1% intervals. We applied a linear stretch to rescale the utilisation distribution predicted values between 0 and 1 according to[55], whereby each pixel value had the minimum UD value subtracted and the result divided by the maximum UD value minus the minimum UD value. For this analysis the scaled UD value was inverted (multiplied by minus 1) so that the hypothesized positive relationship between risk and vigilance could be visualised appropriately. For the categorical variable (for home-range familiarity), we defined the isopleths at $33.\dot{3}$% intervals to explore whether distinct differences between core, frequently used, and boundary areas influenced looking patterns. The number of GPS points for looking focal observations falling within these bounds was relatively similar: core areas – 1302 focal observations, frequently used areas – 1352, boundary areas – 1022.

We used the same methods (as with the utilisation distribution at 1% intervals) to calculate and scale the distribution of within-group encounters during the same period. In this case the time-scaled distance metric was set to 0 to reflect GPS points being collected opportunistically. The subsequent distribution was then scaled (as above) and divided by the scaled UD to produce a layer providing a proxy for spatial probability of an intergroup encounter, offset by home range utilisation, this variable was scaled a further time to ensure all values were between 0 and 1.

To explore whether the study group altered their looking patterns pre-emptively in response to varying spatial risk of encountering leopards, we used the scale integration (see [55]) of the 2nd and 3rd order resource selection function calculated by [44] for leopards utilising the same study area as the study group of baboons. Previously we have used solely the 3rd order RSF to explore pre-emptive vigilance hypotheses in this group[43]. Yet, the scale integration of multiple functions should be an improvement upon this as it incorporates additional environmental information (such as ruggedness and the slope of the landscape) which could have an important role in determining the probability of encountering a leopard. Initially a linear stretch was applied to each RSF to rescale their predicted values between 0 and 1, after which the scale probabilities of each pixel were multiplied (i.e., *P* (2nd order RSF) x *P* (3rd order RSF)), and finally scaled again using the linear stretch equation, resulting in scale integrated RSF layer[55], which represents the spatial probability of a baboon encountering a leopard. All spatial layers are shown figures S11 – S15.

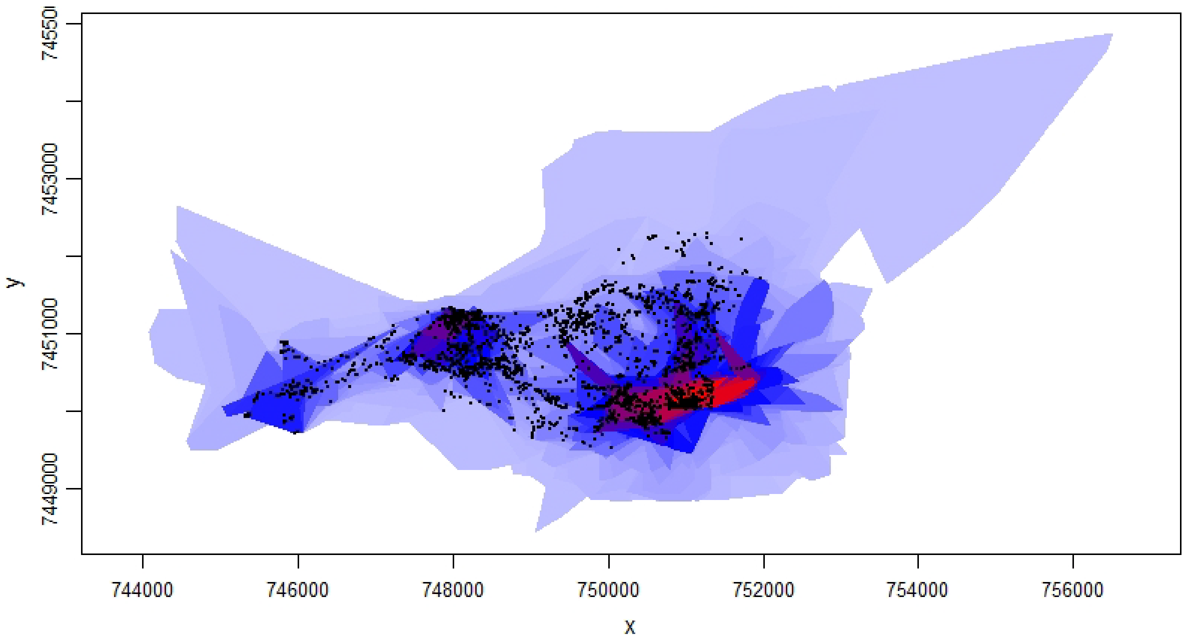

Figure S11. Utilisation distribution for study group calculated from GPS point collected at 20-minute intervals between 2015 and 2019. Light blue represents low use areas and red high-use areas. Black points are the distribution of focal observation used for the looking analysis.

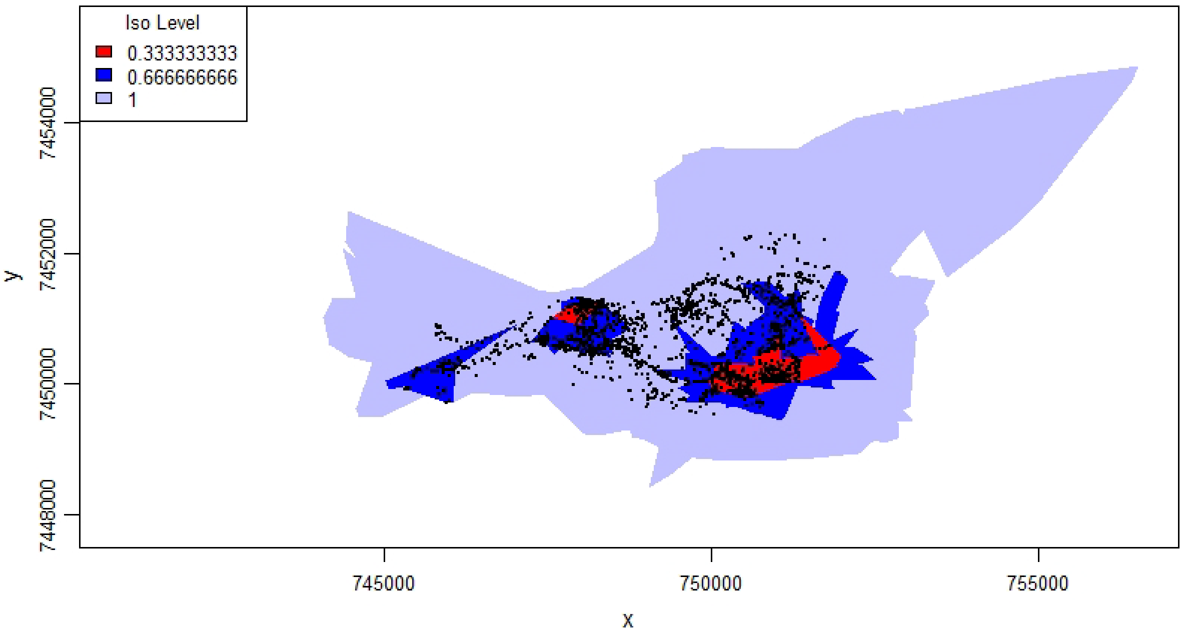

Figure S12. Utilisation distribution for study group calculated from GPS point collected at 20-minute intervals between 2015 and 2019. Light blue represents low use 'boundary' areas (iso level of 1), dark blue represent 'frequently' used areas (iso level of 0.6), and red represents high-use 'core' areas (iso level 0.3). Black points are the distribution of focal observation used for the looking analysis.

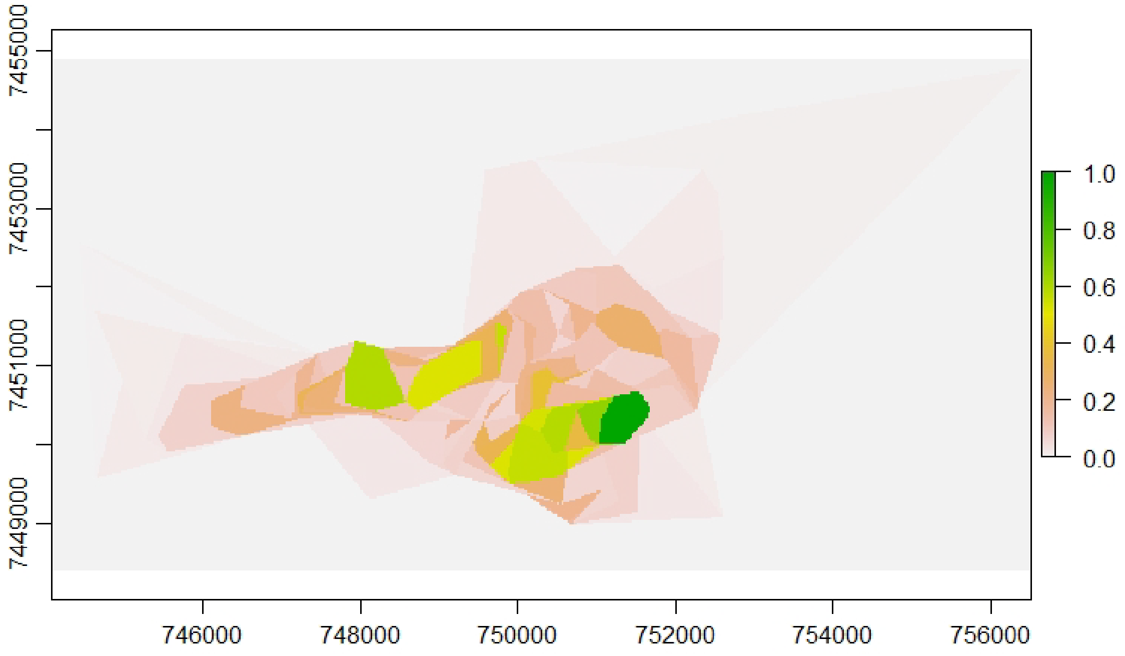

Figure S13. Scaled raster layer representing the distribution of observed encounters between the study group and other groups of baboons.

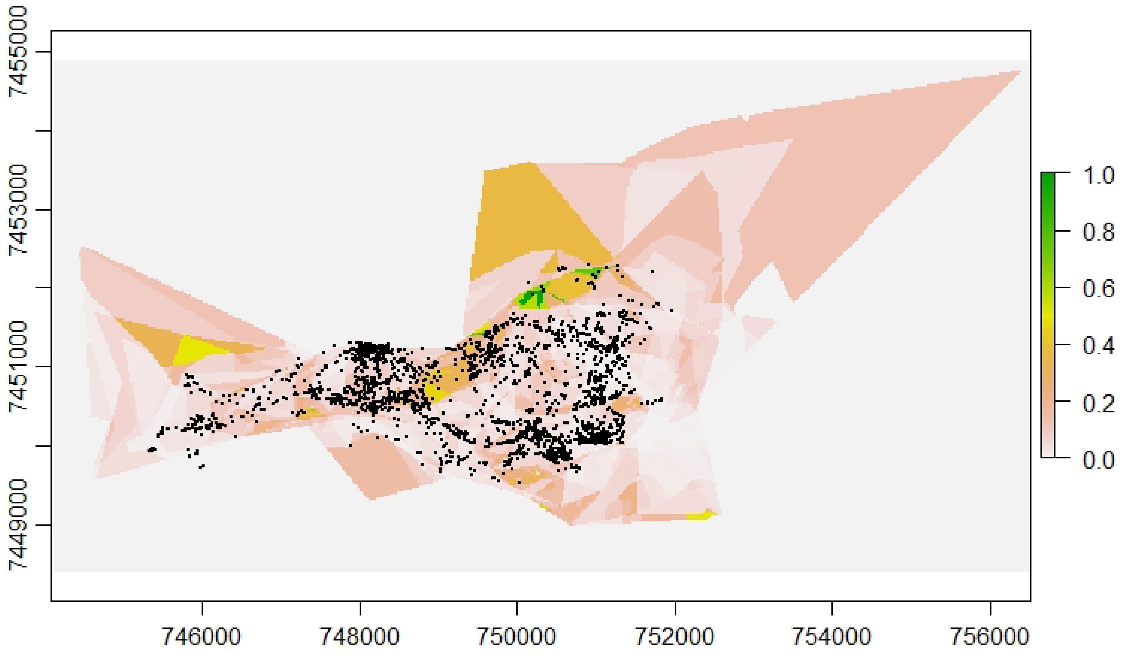

Figure S14. Scaled raster layer representing the probability of encountering another group. Scaled raster layer of distribution of observed encounters between the study group and other groups (Fig S13) divided by a raster of the scaled utilisation distribution (S11).

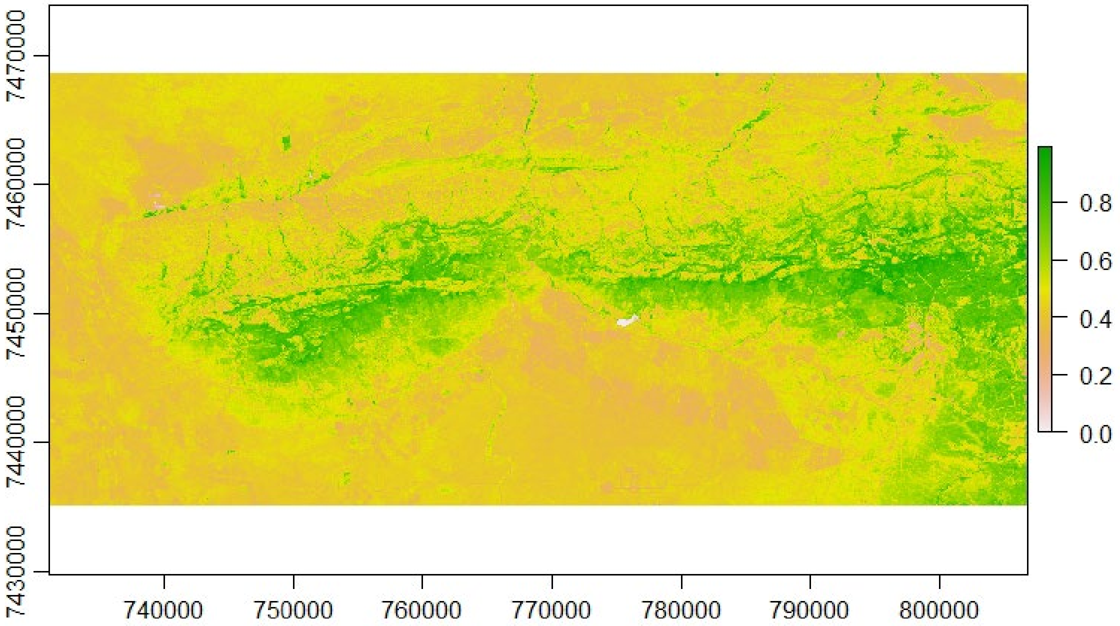

Figure S15. Scale integrated RSF calculated by multiplying each pixel from the 2nd order and 3rd order leopard resource selection functions calculated by [44].

Text S8: Calculating visual tolerance

Visual orientation distance (VOD) was previously found to be distinct amongst individuals and repeatable within each individual, evidence that visual tolerance may be an individual level trait[47]. VOD remained consistent across individuals and the group regardless of whether the approaching observer was familiar or unfamiliar and we found no evidence of habituation or sensitization effects across any temporal level. Nevertheless, to additionally guard against these factors in this study, the model used to calculate visual tolerance estimates (i.e., conditional modes) was updated from[47] to include additional fixed effects that tracked individual trial number per day and group trial number across all individuals per day. We retained the observer identity and observer trial number interaction as both fixed effects and random slopes over individual identity. This interaction was integral to our study design and ensured habituation and sensitization effects were explored effectively across all temporal levels. We removed the fixed effects of neighbour flee first and external factors within 5 minutes from the models used in [47] as they were previously shown not to effect FID in a significant way and we did not want to over parameterize the updated model.

As with[47], the fixed effects of engaged, habitat, height, and number of neighbours within 5 meters were also retained as all were found to be important previously. Date was also included as a random effect crossed with individual identity. Visual orientation distance delay (the difference between start distance and VOD) was included as a fixed effect and random slope over individual identity to control for issues relating to the envelope constraint see[37,56]. In all cases, random slopes were modelled with correlated intercepts to ensure the estimated model captured all levels of by-individual variation. As this study was also focused on social factors inherent to each study animal, we also included dominance rank and age-sex class as fixed effects in the updated model, ensuring these elements of phenotypic variation were accounted for in the individual tolerance estimates [49]. Rank was calculated using the *isi13* function from the *compete* package[57], based on all displacement, supplant, and agonistic dyadic events between April 2017 and April 2018 (n=908), fights between males were excluded as the 'winner' can often be subjective during conflict. Age-sex class was defined according to secondary sexual characteristics (e.g., testes descending/enlarging, sexual swelling, canine eruption) and changes in pelage throughout juvenile development (see Text S2 for full descriptions).

The updated model was fit using the *brm* function from the *brms* package[58] in the R software[59]. Each model was run for six Hamiltonian Markov chains for 15000 iterations, warmup iterations were set to 5000 and adapt_delta to 0.95. All these parameters were set higher than default to allow algorithms to converge efficiently, producing robust posterior samples[58,60]. The model was fit with a Log-normal response distribution and default link function. The Gelman-Rubin convergence diagnostic (Rhat) was equal to 1 in all cases, strongly suggesting accuracy of the response variable to the Log-normal response distribution, i.e., the standard deviation of occurrence points formed around the Log-normal function was minimal. Normal priors (mean = 0, standard deviation = 100) were assigned for population (i.e., fixed) effects within the brm function; the remaining model components were assigned default Student *t* priors (df = 3, mean = 0, scaling factor = 10), apart from the standard deviations of the group-level effects which were constrained to be positive and therefore used a half Student-t prior with the same df, mean, and scaling factor. We compared our observed data to data simulated from the posterior predictive distribution of our models using the pp_check function and were confident our models were able to accurately predict our observed data. The updated model allowed us to extract conditional modes for each individual, these values represented their relative sensitivity to observer approaches and therefore should be an ecologically valid variable to include in analyses exploring looking/vigilance behaviours using direct sampling. For

example, sensitive individuals may glance or increase monitoring effort if the observer is too close or moves during the observation.

Table S29. Updated visual orientation distance model results

**Population-level effects**							
	Estimate	Est.Error	L-95% CI	U-95% CI	Rhat	Bulk_ESS	Tail_ESS
Intercept	1.32	0.2	0.93	1.72	1	21442	33547
VODD	-0.01	0.01	-0.03	0.01	1	41824	44013
Compatibility (Looking)	0.21	0.02	0.17	0.24	1	82948	46341
Compatibility (Not engaged not looking)	0.11	0.02	0.06	0.16	1	87181	48336
Open (Habitat)	0.16	0.02	0.12	0.19	1	93579	44814
Ground (Height)	0.04	0.05	-0.05	0.14	1	91190	48336
Number of neighours	-0.04	0.01	-0.06	-0.03	1	99690	46290
Unfamiliar observer (AB)	-0.24	0.14	-0.52	0.05	1	25433	34299
Individual trial number per observer	0	0.01	-0.02	0.02	1	37100	42669
Group trial number per observation day	-0.01	0	-0.02	0	1	29037	38175
Individual trial number per observation day	0	0.03	-0.05	0.05	1	91368	47158
Dominance rank	0	0	0	0	1	28745	38582
Adolescent males	-0.1	0.15	-0.39	0.18	1	16437	31676
Adolescent females	0.14	0.09	-0.04	0.33	1	14228	26204
Adult females with infants	0.23	0.09	0.04	0.41	1	15390	27741
Adult males	0.15	0.13	-0.11	0.41	1	15639	26900
Juvenile females (J1F)	-0.03	0.12	-0.27	0.21	1	17193	28360
Juvenile males (J1M)	-0.16	0.11	-0.38	0.06	1	13976	28095
Juvenile females (J2F)	-0.06	0.12	-0.29	0.18	1	18648	30249
Juvenile males (J2M)	-0.12	0.12	-0.35	0.11	1	13541	27141
Juvenile males (J3M)	-0.04	0.12	-0.29	0.2	1	14379	28584
Unfamiliar observer (AB) : Trial number per observer	0.01	0.01	-0.01	0.03	1	27018	37502
**Family specific (log-normal)**							
Sigma	0.31	0.01	0.3	0.32	1.00	56100	43987
**Group-level effects**							
Date (58 levels)							
sd(Intercept)	0.1	0.01	0.07	0.13	1.00	17429	31353
Individual identity (69 levels)							
sd(Intercept)	0.18	0.03	0.13	0.25	1.00	18997	32193
sd(VODD)	0.04	0.01	0.03	0.06	1.00	27578	37332
sd(ObserverAB)	0.1	0.04	0.01	0.17	1.00	11854	13081
sd(TrialNo)	0.01	0	0	0.02	1.00	10034	23981
sd(ObserverAB:TrialNo)	0.01	0.01	0	0.02	1.00	7128	18929
cor(Intercept,VODD)	0.6	0.2	0.16	0.91	1.00	15412	27619
cor(Intercept,ObserverAB)	0.33	0.29	-0.3	0.82	1.00	24189	32547
cor(VODD,ObserverAB)	0.25	0.3	-0.39	0.76	1.00	27606	34331
cor(Intercept,TrialNo)	-0.45	0.34	-0.89	0.41	1.00	21043	35075
cor(VODD,TrialNo)	-0.17	0.34	-0.77	0.56	1.00	33161	41968
cor(ObserverAB,TrialNo)	-0.14	0.37	-0.79	0.61	1.00	34430	45452

cor(Intercept,ObserverAB:TrialNo)	0.2	0.34	-0.53	0.77	1.00	24616	35985
cor(VODD,ObserverAB:TrialNo)	0.19	0.33	-0.52	0.76	1.00	39174	37550
cor(ObserverAB,ObserverAB:TrialNo)	-0.03	0.39	-0.71	0.73	1.00	24440	38864
cor(TrialNo,ObserverAB:TrialNo)	-0.29	0.4	-0.88	0.6	1.00	12224	32548

We found no evidence that the study subjects habituated or sensitized across any timeframe as a result of the approaches completed previously (Table S29). Ongoing monitoring of study subject's behavioural responses also validated this (see [47]), providing strong evidence that the methods did not create stress or anxiety in the study subjects. We also found no evidence that dominance rank was a driver of VOD (estimate 0 with upper and lower 95% upper credible intervals both at zero). Most age-sex classes had credible intervals that included zero, suggesting little confidence that age-sex class clearly drove visual orientation distance (see fig S16). However, adult males and females visually oriented slightly quicker than the younger individuals, with adult females with infants having the highest mean conditional effect, however, this may be expected as adult females with infants are likely to be the most risk sensitive animals whilst adult males can include individuals who immigrated from surrounding non-habituated groups, thus their individual tolerances were lower.

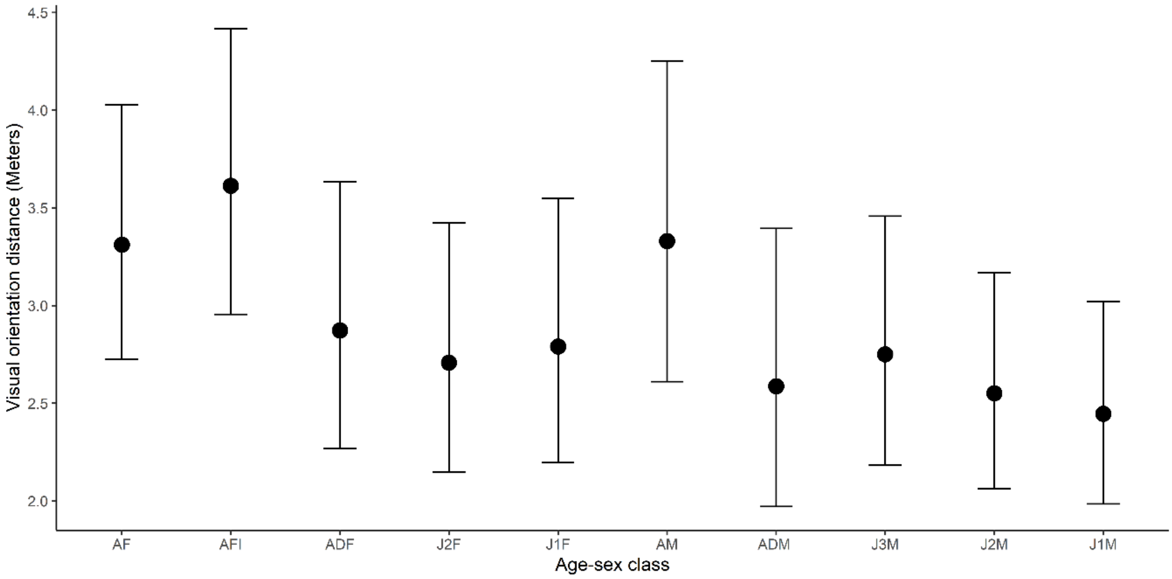

Figure S16. Conditional effects plot for each age-sex class and flight initiation distance. The mean was used as the measure of central tendency, 2.5 and 97.5% percent credible intervals also displayed. From left to right on the x-axis: adult females, adult females with infant, adolescent females, juvenile-2 females, juvenile-1 females, adult males, adolescent males, juvenile-3 males, juvenile-2 males, and juvenile-1 males (see text S2 for descriptions).

The intraclass correlation coefficient (ICC) for the updated model was slightly lower (updated individual identity ICC: 0.26; highest density intervals (HDI) for posterior samples at 95% intervals, 0.14, 0.39) compared to the findings reported in [47] (ICC, 0.38; HDI, 0.24,0.51). ICC estimates the ratio of the variance associated with individual identity effect divided by the total variance, i.e., sum of individual and residual variances, ($VAR_{ind}/VAR_{ind} + VAR_{resid}$), and therefore informs researchers of the degree of variance explained by individual differences, i.e., individual consistency [61].

The sum of individual variances for the updated model was (2113.874) far lower than the sum of the individual variances from the original model (3592.26), suggesting the updated model explained less individual level variance than the previous model. The higher sum of residual variances in the

updated model (5758.474) compared to the original model (5751.353) also suggests that the unexplained variance is lower in the original model compared to the updated one. As a result of these factors the ICC equation produces a lower value for the updated model. Regardless of the decrease in ICC estimate, the HDI parameter values did not include zero, suggesting there was still moderate evidence for repeatability of VOD.

We then extracted the individual conditional modes from the updated model using the *ranef* function in *brms* and performed a Pearson's correlation between the conditional modes from the updated model and the conditional modes from the previous article. Individual tolerance estimates were consistent ($r(67) = .876$, $p < .001$) despite the changes in model structure from [47]. As such, given the similarity in the conditional modes between the models and that the updated model accounted for the phenotypic variation caused by dominance rank and age-sex class, we elected to use the conditional modes derived from the updated model as our individual level visual tolerance estimates in this study, despite the minor decrease in ICC for the updated model.

Consistency across years

To understand whether individual visual tolerance estimates varied between years (and were therefore applicable to the 2018/19 looking analysis) we repeated the procedures outlined in [47] on a subset of 15 individuals (approximately 25% of group members) that were present across 2017, 2018, and 2019. Due to time constraints and results from the previous approach we allowed for up to 4 approaches per individual per sample day, but never sequentially. All individuals received 12 approaches by AA for the 2019 samples.

For the first part of this analysis, we combined the data collected during 2017 and 2018 with the data collected during 2019. The analysis described in [47] was repeated on this dataset. The only changes to this VOD model were that the observer identity and trial number interaction was removed (as only AA completed trials in 2019) and were replaced with individual trial number (for AA specifically) as a fixed effect and random slope over individual identity. Group trial number per day and individual trial number per day were also included as fixed effects to control for habituation and sensitization effects across a number of temporal levels. We also included 'year' as a fixed effect to explore consistency between years across all individuals. We removed the fixed effects of neighbour flee first and external factors within 5 minutes from the models used in [47] as they were previously shown not to effect FID in a significant way and we did not want to over parameterize the model. With 2017 as the reference category, the model estimates for 2018 and 2019 were 0.05 (-0.09, 0.20) and 0.09 (-0.06, 0.23) respectively. In each case estimates were close to zero with credible intervals overlapping zero, providing strong support for there being no effect of year on FID.

In addition, we ran a 2019 model using the same analytical framework as described above based only on 2019 FID data from the sample of 15 individuals. The only changes to the model was the removal of the covariate 'year'. We then extracted the individual conditional modes from the model and performed a Pearson's correlation between the 2019 conditional modes and the conditional modes from the updated model (from the main text - 2017/18 data). Results supported that tolerance estimates were consistent across years ($r(13) = .77$, $p < .001$), as such we felt confident utilising the data collected during 2017/2018 for all individuals in the updated model. Conditional modes extracted from the updated model were originally on the spectrum whereby highly tolerant animals had low/negative estimates and highly intolerant animals had high/positive estimates; therefore, tolerance was multiplied by minus 1 to reverse the scale for more logical inference in this study.

Text S9. Model checks

We assessed model fits via graphical checks, firstly by examining trace plots to ensure good mixing and convergence of multiple chains, and secondly, by comparing our observed data to data simulated from the posterior predictive distribution of our models using the pp_check and ppc_stat functions (*brms* and *bayesplot* packages). The simulated data from each of our models generated data that captured the vast majority of values in our observed response distributions and did not fail to account for large proportions of zeroes or censored values in our observed datasets[58,62]. The Rhat values of all models were less than 1.01, indicating accuracy of the response variables with regard to the response distributions and excellent chain convergences. In addition, the bulk effective sample sizes (ESS) were more than 100 times the number of chains (minimum advised threshold) in all cases, with tail ESS also being well sampled in all cases. Together these factors highlight that all models produced high estimation accuracy, including at the tails of the distribution[58]. Finally, we checked for multicollinearity using the check_collinearity function from the *Performance* package[63], and all predictors produced low variance inflation factors (VIF), strong evidence that multicollinearity was not an issue. The only exception was for the specific behaviours model (see Table 1, model 5) where the inclusion of 'resting' created high VIF correlations. Although this does not affect prediction, it can inflate standard errors and bias interpretations of model estimates; we therefore removed resting from these models. We do however, still present the estimates and credible intervals for resting activities in the results (from a resting-only model and the specific behaviours model with multicollinearity issues that included resting).

References

1. Creel, S., Schuette, P. & Christianson, D. Effects of predation risk on group size, vigilance, and foraging behavior in an African ungulate community. *Behavioral Ecology* **25**, 773–784 (2014).

2. Ota, K. Fight, fatigue and flight: Narrowing of attention to a threat compensates for decreased anti-predator vigilance. *Journal of Experimental Biology* **221**, (2018).

3. Busia, L., Schaffner, C. M. & Aureli, F. Watch out or relax: conspecifics affect vigilance in wild spider monkeys (Ateles geoffroyi). *Behaviour* **153**, 107–124 (2016).

4. Kutsukake, N. Conspecific influences on vigilance behavior in wild chimpanzees. *Int J Primatol* **28**, 907–918 (2007).

5. Fernández-Juricic, E. & Beauchamp, G. An experimental analysis of spatial position effects on foraging and vigilance in brown-headed cowbird flocks. *Ethology* **114**, 105–114 (2008).

6. Öst, M., Jaatinen, K. & Steele, B. Aggressive females seize central positions and show increased vigilance in brood-rearing coalitions of eiders. *Anim Behav* **73**, 239–247 (2007).

7. Bednekoff, P. A. & Blumstein, D. T. Peripheral obstructions influence marmot vigilance : integrating observational and experimental results. *Behavioral Ecology* **20**, 1111–1117 (2009).

8. Lima, S. L. Vigilance while feeding and its relation to the risk of predation. *J Theor Biol* **124**, 303–316 (1987).

9. Cowlishaw, G. The role of vigilance in the survival and reproductive strategies of Desert Baboons. *Behaviour* **135**, 431–452 (1998).

10. Fuller, R. A., Bearhop, S., Metcalfe, N. B. & Piersma, T. The effect of group size on vigilance in Ruddy Turnstones Arenaria interpres varies with foraging habitat. *Ibis* **155**, 246–257 (2013).

11. Campos, F. A. & Fedigan, L. M. Spatial ecology of perceived predation risk and vigilance behavior in white-faced capuchins. *Behavioral Ecology* **25**, 477–486 (2014).

12. Steenbeek, R., Piek, R. C., van Buul, M. & van Hooff, J. A. R. A. M. Vigilance in wild Thomas's langurs (Presbytis thomasi): The importance of infanticide risk. *Behav Ecol Sociobiol* **45**, 137–150 (1999).

13. McNelis, N. L. & Boatright-Horowitz, S. L. Social monitoring in a primate group: The relationship between visual attention and hierarchical ranks. *Anim Cogn* **1**, 65–69 (1998).

14. Cameron, E. Z. & Du Toit, J. T. Social influences on vigilance behaviour in giraffes, Giraffa camelopardalis. *Anim Behav* **69**, 1337–1344 (2005).

15. Lima, S. L. Initiation and Termination of Daily Feeding in Dark-Eyed Juncos: Influences of Predation Risk and Energy Reserves. *Oikos* **53**, 3–11 (1988).

16. Burger, J. & Gochfeld, M. Effects of ecotourists on bird behaviour at Loxahatchee National Wildlife Refuge, Florida. *Environ Conserv* **25**, 13–21 (1998).

17. Brivio, F., Grignolio, S., Brambilla, A. & Apollonio, M. Intra-sexual variability in feeding behaviour of a mountain ungulate: size matters. *Behav Ecol Sociobiol* **68**, 1649–1660 (2014).

18. Monclus, R. & Rodel, H. G. Influence of Different Individual Traits on Vigilance Behaviour in European Rabbits. *Ethology* **115**, 758–766 (2009).

19. Lazarus, J. & Inglis, I. R. The Breeding Behaviour of the Pink-Footed Goose: Parental Care and Vigilant Behaviour during the Fledging Period. *Behaviour* **65**, 62–88 (1978).

20. Li, Z., Jiang, Z. & Beauchamp, G. Vigilance in Przewalski's gazelle: effects of sex, predation risk and group size. *J Zool* **277**, 302–308 (2009).

21. Treves, A., Drescher, A. & Snowdon, C. T. Maternal watchfulness in black howler monkeys (Alouatta pigra). *Ethology* **109**, 135–146 (2003).

22. Onishi, K. & Nakamichi, M. Maternal Infant Monitoring in a Free-ranging Group of Japanese Macaques (Macaca fuscata). *Int J Primatol* **32**, 209–222 (2011).

23. Baldellou, M. & Henzi, P. S. Vigilance, predator detection and the presence of supernumerary males in vervet monkey troops. *Anim Behav* **43**, 451–461 (1992).

24. Burger, J. & Gochfeld, M. Effects of group size and sex on vigilance in ostriches (Stuthio camelus): Antipredator strategy or mate competition? *Ostrich: Journal of African Ornithology* **59**, 14–20 (1988).

25. Nowak, K., Le Roux, A., Richards, S. A., Scheijen, C. P. J. & Hill, R. A. Human observers impact habituated samango monkeys' perceived landscape of fear. *Behavioral Ecology* **25**, 1199–1204 (2014).

26. Boinski, S. U. E. *et al.* Are vigilance, risk from avian predators and group size consequences of habitat structure? A comparison of three species of squirrel monkey (Saimiri oerstedii, S. boliviensis, S. sciureus). *Behaviour* **139**, 1421–1467 (2003).

27. Fragaszy, D. Sex and age differences in the organisation of behaviour in wedge-capped capuchins, Cebus olivaceus. *Behavioral Ecology* **1**, 81–94 (1990).

28. Gosselin-Ildari, A. D. & Koenig, A. The Effects of Group Size and Reproductive Status on Vigilance in Captive Callithrix jacchus. *Am J Primatol* **74**, 613–621 (2012).

29. Treves, A., Drescher, A. & Ingrisano, N. Vigilance and aggregation in black howler monkeys (Alouatta pigra). *Behav Ecol Sociobiol* **50**, 90–95 (2001).

30. Treves, A. Within-group vigilance in red colobus and redtail monkeys. *Am J Primatol* **48**, 113–126 (1999).

31. Gaynor, K. M. & Cords, M. Antipredator and social monitoring functions of vigilance behaviour in blue monkeys. *Anim Behav* **84**, 531–537 (2012).

32. Kutsukake, N. The context and quality of social relationships affect vigilance behaviour in wild chimpanzees. *Ethology* **112**, 581–591 (2006).

33. Cords, M. Predator Vigilance Costs of Allogrooming in Wild Blue Monkeys. *Behaviour* **132**, 559–569 (1995).

34. Cowlishaw, G. *et al.* A simple rule for the costs of vigilance: empirical evidence from a social forager. *Proceedings of the Royal Society B: Biological Sciences* **271**, 27–33 (2004).

35. Blanchard, P. & Fritz, H. Induced or routine vigilance while foraging. *Oikos* **116**, 1603–1608 (2007).

36. Hall, K. R. L. Numerical Data, Maintenance Activities and Locomotion of the Wild Chacma Baboon, Papio Ursinus. *Proceedings of the Zoological Society of London* **139**, 181–220 (1962).

37. Allan, A. T. L. & Hill, R. A. What have we been looking at? A call for consistency in studies of primate vigilance. *Am J Phys Anthropol* **165**, 4–22 (2018).

38. Teichroeb, J. A. & Sicotte, P. Cost-free vigilance during feeding in folivorous primates? Examining the effect of predation risk, scramble competition, and infanticide threat on vigilance in ursine colobus monkeys (Colobus vellerosus). *Behav Ecol Sociobiol* **66**, 453–466 (2012).

39. Treves, A. Theory and method in studies of vigilance and aggregation. *Anim Behav* **60**, 711–722 (2000).

40. Suzuki, M. & Sugiura, H. Effects of proximity and activity on visual and auditory monitoring in wild Japanese macaques. *Am J Primatol* **73**, 623–631 (2011).

41. Watts, D. P. A preliminary study of selective visual attention in female mountain gorillas (Gorilla gorilla beringei). *Primates* **39**, 71–78 (1998).

42. Pannozzo, P. L., Phillips, K. A., Haas, M. E. & Mintz, E. M. Social monitoring reflects dominance relationships in a small captive group of brown capuchin monkeys (Cebus apella). *Ethology* **113**, 881–888 (2007).

43. Ayers, A. M., Allan, A. T. L. & Hill, R. A. Foraging in fear: spatial variation in range use, vigilance, and perceived risk in chacma baboons (Papio ursinus). *In review*.

44. Ayers, A. M. The behavioural ecology and predator-prey interactions of leopards (Panthera pardus) and chacma baboons (Papio ursinus) in an Afromontane environment. (Durham University, 2019).

45. MacIntosh, A. J. J. & Sicotte, P. Vigilance in ursine black and white colobus monkeys (colobus vellerosus): An examination of the effects of conspecific threat and predation. *Am J Primatol* **71**, 919–927 (2009).

46. Treves, A. & Brandon, K. Tourist impacts on the behavior of black howler monkeys (Alouatta pigra) at Lamanai, Belize. in *Commensalism and conflict: the human-primate interface* 147–167 (2005).

47. Allan, A. T. L., Bailey, A. L. & Hill, R. A. Habituation is not neutral or equal: Individual differences in tolerance suggest an overlooked personality trait. *Sci Adv* **6**, eaaz0870 (2020).

48. Allan, A. T. L., Bailey, A. L. & Hill, R. A. Consistency in the flight and visual orientation distances of habituated chacma baboons after an observed leopard predation. Do flight initiation distance methods always measure perceived predation risk? *Ecol Evol* **11**, 15404–15416 (2021).

49. Allan, A. T. L., White, A. F. & Hill, R. A. Intolerant baboons avoid observer proximity, creating biased inter-individual association patterns. *Sci Rep* **12**, 8077 (2022).

50. Caine, N. G. & Marra, S. L. Vigilance and social organization in two species of primates. *Anim Behav* **36**, 897–904 (1988).

51. Haude, R. H., Graber, J. G. & Farres, A. G. Visual observing by rhesus monkeys: Some relationships with social dominance rank. *Anim Learn Behav* **4**, 163–166 (1976).

52. Keverne, E. B., Leonard, R. A., Scruton, D. M. & Young, S. K. Visual monitoring in social groups of talapoin monkeys (Miopithecus talapoin). *Anim Behav* **26**, 933–944 (1978).

53. Lyons, A. J., Turner, W. C. & Getz, W. M. Home range plus: a space-time characterization of movement over real landscapes. *Mov Ecol* **1**, 2 (2013).

54. Getz, W. M. *et al.* LoCoH: Nonparameteric Kernel methods for constructing home ranges and utilization distributions. *PLoS One* **2**, e207 (2007).

55. Pitman, R. T. *et al.* Cats, connectivity and conservation: incorporating data sets and integrating scales for wildlife management. *Journal of Applied Ecology* **54**, 1687–1698 (2017).

56. Bonnot, N. C. *et al.* Stick or twist: roe deer adjust their flight behaviour to the perceived trade-off between risk and reward. *Anim Behav* **124**, 35–46 (2017).

57. Curley, J. P. compete: Analyzing Social Hierarchies: R package version 0.1. https://www.rdocumentation.org/packages/compete/versions/0.1 (2016).

58. Bürkner, P.-C. brms : An R Package for Bayesian Multilevel Models Using Stan. *J Stat Softw* **80**, 1–28 (2017).

59. R Core Team. R: A language and environment for statistical computing. Version 4.1.1. *R Foundation for Statistical Computing, Vienna, Austria* https://www.r-project.org/ (2021).

60. McElreath, R. *Statistical Rethinking 2: A Bayesian Course with Examples in R and Stan*. (2019).

61. Houslay, T. M. & Wilson, A. J. Behavioral Ecology Avoiding the misuse of BLUP in behavioural ecology. *Behavioral Ecology* **28**, 948–952 (2017).

62. Gabry, J., Simpson, D., Vehtari, A., Betancourt, M. & Gelman, A. Visualization in Bayesian workflow. *J R Stat Soc Ser A Stat Soc* **182**, 389–402 (2019).

63. Lüdecke, D., Ben-Shachar, M., Patil, I., Waggoner, P. & Makowski, D. performance: An R Package for Assessment, Comparison and Testing of Statistical Models. *J Open Source Softw* **6**, 3139 (2021).
