# Supplementary material for: Behavioural compatibility, not fear, best predicts the looking patterns of chacma baboons
Source: Commun Biol. 2024 Aug 12;7:980. doi: 10.1038/s42003-024-06657-w (PMC11319805; doi:10.1038/s42003-024-06657-w)
Supplement: Supplementary file 3 — Reporting summary [file 42003_2024_6657_MOESM3_ESM.pdf]

nature portfolio

Corresponding author(s): Andrew Allan

Last updated by author(s): Jul 5, 2024

Reporting Summary

Nature Portfolio wishes to improve the reproducibility of the work that we publish. This form provides structure for consistency and transparency in reporting. For further information on Nature Portfolio policies, see our Editorial Policies and the Editorial Policy Checklist.

Statistics

For all statistical analyses, confirm that the following items are present in the figure legend, table legend, main text, or Methods section.

n/a	Confirmed	
☐	☒	The exact sample size (*n*) for each experimental group/condition, given as a discrete number and unit of measurement
☐	☒	A statement on whether measurements were taken from distinct samples or whether the same sample was measured repeatedly
☐	☒	The statistical test(s) used AND whether they are one- or two-sided<br>*Only common tests should be described solely by name; describe more complex techniques in the Methods section.*
☐	☒	A description of all covariates tested
☐	☒	A description of any assumptions or corrections, such as tests of normality and adjustment for multiple comparisons
☐	☒	A full description of the statistical parameters including central tendency (e.g. means) or other basic estimates (e.g. regression coefficient) AND variation (e.g. standard deviation) or associated estimates of uncertainty (e.g. confidence intervals)
☐	☒	For null hypothesis testing, the test statistic (e.g. *F*, *t*, *r*) with confidence intervals, effect sizes, degrees of freedom and *P* value noted<br>*Give P values as exact values whenever suitable.*
☐	☒	For Bayesian analysis, information on the choice of priors and Markov chain Monte Carlo settings
☐	☒	For hierarchical and complex designs, identification of the appropriate level for tests and full reporting of outcomes
☐	☒	Estimates of effect sizes (e.g. Cohen's *d*, Pearson's *r*), indicating how they were calculated

Our web collection on statistics for biologists contains articles on many of the points above.

Software and code

Policy information about availability of computer code

Data collection	No software used
Data analysis	All data analysis was conducted in R.

For manuscripts utilizing custom algorithms or software that are central to the research but not yet described in published literature, software must be made available to editors and reviewers. We strongly encourage code deposition in a community repository (e.g. GitHub). See the Nature Portfolio guidelines for submitting code & software for further information.

Data

Policy information about availability of data

All manuscripts must include a data availability statement. This statement should provide the following information, where applicable:

- Accession codes, unique identifiers, or web links for publicly available datasets
- A description of any restrictions on data availability
- For clinical datasets or third party data, please ensure that the statement adheres to our policy

Raw data has been uploaded to figshare

Research involving human participants, their data, or biological material

Policy information about studies with human participants or human data. See also policy information about sex, gender (identity/presentation), and sexual orientation and race, ethnicity and racism.

Reporting on sex and gender	N/A
Reporting on race, ethnicity, or other socially relevant groupings	N/A
Population characteristics	N/A
Recruitment	N/A
Ethics oversight	N/A

Note that full information on the approval of the study protocol must also be provided in the manuscript.

Field-specific reporting

Please select the one below that is the best fit for your research. If you are not sure, read the appropriate sections before making your selection.

☐ Life sciences ☐ Behavioural & social sciences ☒ Ecological, evolutionary & environmental sciences

For a reference copy of the document with all sections, see nature.com/documents/nr-reporting-summary-flat.pdf

Ecological, evolutionary & environmental sciences study design

All studies must disclose on these points even when the disclosure is negative.

Study description	This study used direct observations of wild, habituated chacma baboons (Papio ursinus grisepes) to record data on looking patterns across different social and ecological contexts.
Research sample	This data was collected on all non-infant individuals (54-59 observations per individual, 3676 total observations). The group were habituated for research purposes in 2005 and contained 80 individuals at the start of the study, increasing to 92 individuals by the end due to births (no permanent immigrations took place). In total, sixty-five individuals were used for this analysis, representing all non-infant individuals present at the start of this study
Sampling strategy	30-second continuous focal sampling was used to record the temporal organisation of looking behaviours using a high-definition video camera (Panasonic HC-W580 Camcorder). Each observation day was split into four seasonally adjusted time-periods that each accounted for 25% of the day length.
Data collection	AA used Media Player Classic (MPC-HC: Guliverkli project) to slow down and extract precise looking bout lengths from videos (video skip length could be reduced to 4 hundredths of a second). A looking bout began when the focal animal's eyes were open, and its line of vision extended beyond its hands and the substrate, animal, or object its hands were in contact with. At the beginning and end of the focal AA also recorded the number and identity of all neighbours within 5 meters of the focal animal, the estimated visibility (percentage) to 5 meters in all directions from the focal animal, and the distance between the focal animal and the observer. These values were then averaged at the analysis stage. At the end of the focal AA also assessed the habitat type and spatial position for the majority of the focal observation. Specific behavioral information was extracted from video footage during the data processing stage. Time to most various events (e.g., inter-group encounters, predator encounters, within-group aggressions) was updated throughout the day and this information also included as covariates during the analysis.
Timing and spatial scale	The focal animal sampling for looking patterns was conducted at Lajuma Research Centre, western Soutpansberg Mountains, South Africa (central coordinates S29.44031°, E23.02217°) between May 2018 and July 2019. Ranging and interaction data used for utilisation distributions and risk landscapes were collected between February 2015 and July 2019 at the same field site.
Data exclusions	Focal observations were deemed successful if at least 25 seconds of footage had at least 50% of the animal's face in view. Observations were aborted or discarded if more than 50% of the focal animal's face was out of sight for more than 5 seconds. In these scenarios AA would then adjust position and try to restart the focal observation, a process that was repeated a maximum of three times before moving to another individual from the list. The individual receiving the aborted focal would then be reintegrated at the end of the list. Animals that disappeared during the study period were removed from the main focal looking analysis, but their influence on focal animals (i.e., as a neighbour) was still explored for the periods they were still in the group.
Reproducibility	N/A - This was an observational study on wildlife that could be repeated using the same methods.
Randomization	A 'randomly' generated observation list was created and focal individuals were then selected pseudo-randomly from this list by sampling the first individual encountered from the top 15 identities on the list (approximately 20% of original group-size). An information-theoretic approach was used to identify all realistic biological hypotheses that may explain looking patterns in wild,

habituated animals. We therefore collected data on numerous social (e.g., number of social threats within 5m, number of neighbours within 5m, time since within-group aggression), environmental (e.g., visibility, habitat type), ecological (e.g., spatial risk of encountering leopards or other groups), behavioral (e.g., specific feeding task and rate, posture), and methodological (e.g., observer tolerance and proximity) confounding variables, which were then included in a range of models designed to test specific hypotheses. These models were assessed for their predictive precision via Bayesian R2 estimates and stacking of posterior distributions, the results then revealed which factors were the best predictors of looking patterns.

Blinding: No blinding as data collected via direct observations and coded from video-footage by AA.

Did the study involve field work? ☒ Yes ☐ No

Field work, collection and transport

Field conditions: The area was designated Afro-montane mist-belt community and contained a diverse range of natural habitats varying in plant species composition, canopy height, and foliage density. Most of the study area was classified as private nature reserve, but agricultural practices and habitat modification occurred in areas adjacent to the study group's core home range. The major predator of baboons was leopards (Panthera pardus). Temperature and rainfall was highly variable across seasons, from very dry and hot months (>35C) to cold and rainy conditions (<5C and ~65mm of rainfall).

Location: Lajuma Research Centre, western Soutpansberg Mountains, South Africa (central coordinates S29.44031°, E23.02217°).

Access & import/export: We received research permission from the Limpopo Province Department of Economic Development and Tourism (Permit No. ZA/LP/81996). This research was also approved by the Durham University Animal Welfare Ethical Review Board.

Disturbance: All researchers that observed these habituated baboons received training and protocols to maintain human/animal safety through the Primate and Predator Project. This included observing animals with binoculars from a distance, avoiding direct eye contact and any other potentially disturbing behaviors to minimize observer bias and potential stress to animals. In addition, we previously quantified the tolerance each baboon had towards observers, allowing AA to select observation distances that minimized 'observer effects'. In addition, to check this was successfully achieved, we included to 'observer effects' models in our analysis that specifically explored whether observer distance and movement interacted with tolerance levels to influence the looking patterns of focal individuals - we found no support for these hypotheses, suggesting we successfully minimized 'observer effects'.

Reporting for specific materials, systems and methods

We require information from authors about some types of materials, experimental systems and methods used in many studies. Here, indicate whether each material, system or method listed is relevant to your study. If you are not sure if a list item applies to your research, read the appropriate section before selecting a response.

Materials & experimental systems

n/a	Involved in the study	
☒	☐	Antibodies
☒	☐	Eukaryotic cell lines
☒	☐	Palaeontology and archaeology
☐	☒	Animals and other organisms
☒	☐	Clinical data
☒	☐	Dual use research of concern
☒	☐	Plants

Methods

n/a	Involved in the study	
☒	☐	ChIP-seq
☒	☐	Flow cytometry
☒	☐	MRI-based neuroimaging

Animals and other research organisms

Policy information about studies involving animals; ARRIVE guidelines recommended for reporting animal research, and Sex and Gender in Research

Laboratory animals: Study did not involve laboratory animals

Wild animals: This data was collected on all non-infant individuals (54-59 observations per individual, 3676 total observations). The group were habituated for research purposes in 2005 and contained 80 individuals at the start of the study, increasing to 92 individuals by the end due to births (no permanent immigrations took place). In total, sixty-five individuals were used for this analysis, representing all non-infant individuals present at the start of this study. No individuals were captured or killed by researchers during the study period. After the study was completed, formal observations were no longer conducted on the group, but local researchers confirmed the group is still behaving/ranging normally.

Reporting on sex: Age-sex class and reproductive information was collected as certain factors can change an individual's likelihood of predation or perception of risk (e.g., being heavily pregnant or in consortship with a male). These factors were tested for in our analysis. Age-sex

	class was determined via assessment of baboon's secondary sexual characteristics (e.g., canine length, testes size, swelling size).
Field-collected samples	Study did not involve samples collected from the field.
Ethics oversight	We received research permission from the Limpopo Province Department of Economic Development and Tourism (Permit No. ZA/LP/81996). This research was also approved by the Durham University Animal Welfare Ethical Review Board. This was a fully observational study, but to further safeguard against disturbance we developed a research theme concerning human tolerance, which we tested for in this study.

Note that full information on the approval of the study protocol must also be provided in the manuscript.

Plants

Seed stocks	N/A
Novel plant genotypes	N/A
Authentication	N/A
